# Network meta-analysis on efficacy and safety of different biologics for ulcerative colitis

**DOI:** 10.1186/s12876-023-02938-6

**Published:** 2023-10-06

**Authors:** Xinqiao Chu, Yaning Biao, Chengjiang Liu, Yixin Zhang, Chenxu Liu, Ji-zheng Ma, Yufeng Guo, Yaru Gu

**Affiliations:** 1 Guang’anmen Hospital, China Academy of Chinese Medical Sciences - No.5, Beixian Pavilion, Xicheng District, Beijing, 100053 China; 2 School of Pharmacy, Hebei University of Chinese Medicine, 326 New Shinan Road, Qiaoxi District, Shijiazhuang, Hebei 050091 China; 3 Department of General Medicine, Affiliated Anqing First People’s Hospital of Anhui Medical University, Anqing, Anhui China; 4 School of Basic Medicine, Hebei University of Chinese Medicine, Shijiazhuang, Hebei 050200 China

**Keywords:** Ulcerative colitis, Biologics, Network meta-analysis, Systematic review

## Abstract

**Background:**

Therapeutic options for ulcerative colitis (UC) have increased since the introduction of biologics a few decades ago. Due to the wide range of biologics available, physicians have difficulty in selecting biologics and do not know how to balance the best drug between clinical efficacy and safety. This study aimed to compare the efficacy and safety of biologics in treating ulcerative colitis.

**Methods:**

In this study, eight electronic databases (PubMed, Web of Science, Cochrane, Embase, Sinomed, China National Knowledge Infrastructure, Chongqing VIP Information, and WanFang Data) were searched to collect eligible studies without language restrictions. Retrieved 1 June 2023, from inception. All articles included in the mesh analysis are randomised controlled trials (RCTs). The inclusion of drugs for each outcome was ranked using a curved surface under cumulative ranking (SUCRA). Higher SUCRA scores were associated with better outcomes, whereas lower SUCRA scores were associated with better safety. This study has registered with PROSPERO, CRD42023389483.

**Results:**

Induction Therapy: Among the biologic therapies evaluated for induction therapy, vedolizumab demonstrated the highest efficacy in achieving clinical remission (OR vs daclizumab, 9.09; 95% CI, 1.01–81.61; SUCRA 94.1) and clinical response. Guselkumab showed the lowest risk of recurrence of UC (SUCRA 94.9%), adverse events resulting in treatment discontinuation (SUCRA 94.8%), and serious infections (SUCRA 78.0%). Maintenance Therapy: For maintenance therapy, vedolizumab ranked highest in maintaining clinical remission (OR vs mesalazine 4.36; 95% CI, 1.65–11.49; SUCRA 89.7) and endoscopic improvement (SUCRA 92.6). Infliximab demonstrated the highest efficacy in endoscopic improvement (SUCRA 92.6%). Ustekinumab had the lowest risk of infections (SUCRA 92.9%), serious adverse events (SUCRA 91.3%), and serious infections (SUCRA 67.6%).

**Conclusion:**

Our network meta-analysis suggests that vedolizumab is the most effective biologic therapy for inducing and maintaining clinical remission in UC patients. Guselkumab shows promise in reducing the risk of recurrence and adverse events during induction therapy. Infliximab is effective in improving endoscopic outcomes during maintenance therapy. Ustekinumab appears to have a favorable safety profile. These findings provide valuable insights for clinicians in selecting the most appropriate biologic therapy for UC patients.

**Supplementary Information:**

The online version contains supplementary material available at 10.1186/s12876-023-02938-6.

## Introduction

Ulcerative colitis (UC), being a chronic inflammatory disease affecting the digestive system, is characterized by several symptoms that include diarrhea, fever, fecal mucus and bleeding, acute abdominal pain, weight loss and fatigue [1]. The prolonging of these symptoms often leads to increased anxiety, depression, and reduced quality of life among UC patients. While the global prevalence of UC is evolving, the disease's prevalence is unquestionably on the rise [2–4].

Impaired intestinal mucosal barrier function is recognized as a key contributor to the pathogenesis of ulcerative colitis. Dysregulation of the intestinal environment may lead to augmented intestinal mucosal permeability, activation of macrophages and antigen delivery cells, and consequent inflammatory responses as invasive monocytes differentiate into macrophages, releasing pro-inflammatory cytokines TNF-α, IL-6, IL-12 and IL-23 [5]. Monoclonal antibody treatments for UC typically aim to reduce inflammatory responses in the gut, including inhibiting the expression of pro-inflammatory cytokine and suppressing the immune responses, such as TNF-α monoclonal antibodies and IL-12 / IL-23 antagonists.

As per the prevailing UC treatment guidelines, the suggested course of action involves the implementation of biologics, such as infliximab, adalimumab, golimumab, vedolizumab, ustekinumab, or tofacitinib, in patients with mild-to-severe UC who do not respond well to conventional treatment or are unable to tolerate it [6]. For patients already undergoing high-dose mesalazine maintenance therapy, or afflicted with corticosteroid-dependency or refractory treatment, upgrades to thiopurine, anti-TNF therapy, vedolizumab, or tofacitinib should be taken into consideration [7].

In recent years, there has been a growing interest in the use of biological agents for the treatment of ulcerative colitis (UC). Among these agents, ustekinumab, vedolizumab, and infliximab have received significant attention and have been extensively studied for their therapeutic effects [8]. Previous studies have shown that these agents have comparable efficacy in terms of achieving managed clinical response, sustained clinical response, and mucosal healing. In a meta-evaluation of ustekinumab for UC, it was found that this agent has demonstrated efficacy and safety in both randomized controlled trials (RCTs) and real clinical practice [9]. The most common adverse event reported with ustekinumab was infection, with rates of 34% and 41% in the ustekinumab and adalimumab groups, respectively [10]. Another study by Moens et al. reported similar findings, with eight events of infections in the ustekinumab group and ten events in the adalimumab group [11]. Vedolizumab, another biological agent, has shown a higher clinical response survival rate compared to adalimumab and infliximab in patients with UC who have not been exposed to biologics [12]. To evaluate the efficacy and safety of various biologics, including infliximab, ustekinumab, vedolizumab, and others, several double-blind, randomized, and placebo-controlled clinical trials have been conducted [13–15]. However, there is still a lack of comprehensive studies comparing the efficacy and safety of these agents.

Moreover, with the increasing diversity in mechanisms of action, the promptness of onset of efficacy has become an important factor for clinicians and patients when selecting treatment options. In situations where direct comparisons are not feasible, indirect comparison through grid meta-analysis can be a useful tool for decision-making purposes. Therefore, we conducted an assessment of the efficacy and safety of a range of biologics in patients with UC, based on the available RCTs. The aim of this study was to determine which biologics exhibit optimal therapeutic potential and can assist clinicians in selecting evidence-based protocols for the management of patients with UC.

## Methods

In line with the Preferred Reporting Items for Systematic Reviews and Meta-Analyses for Network Meta-Analyses (PRISMANMA) guidelines [16], we undertook a comprehensive exploration and network meta-analysis (Supplementary Table 1). In addition, our study was registered with PROSPERO, and assigned the unique registration number CRD42023389483.

### Search strategy

A comprehensive search for eligible studies was conducted using numerous online electronic databases, including PubMed, Science, Cochrane, Embase, Sinomed, China National Knowledge Infrastructure, Chongqing VIP Information, and Wan Fang Data. This search was conducted until 1 June 2023. The search was performed using a particular set of keywords and topics (Supplementary Table 2). These included terms such as “infliximab”, “etrolizumab”, “adalimumab”, “vedolizumab”, “ustekinumab”, “cobitolimod”, “PF-00547659”, “eldelumab”, “golimumab”, “BMS-936557”, “basiliximab”, “visilizumab”, “daclizumab”, and “ulcerative colitis”.

### Inclusion and exclusion criteria

#### Inclusion criteria

This study focused on randomized controlled trials involving adult patients diagnosed with ulcerative colitis who were aged 18 years or older. The primary objective was to compare different biological agents administered at market-approved doses against one another or a control group. The control group was defined as either a placebo, conventional drugs or other biologics, which were used as a comparative measure.

#### Exclusion criteria

In order to maintain the coherence and integrity of the study design, studies falling within any of the following categories may be excluded: repeat publications, animal or in vitro testing, case reports, summaries, meta-analyses, letters to the editor, and meeting summaries. Furthermore, the full text of each study must provide sufficient data on efficacy and safety to be considered for inclusion. These criteria are essential for ensuring the selection and evaluation of reliable and valid research data.

### Outcome measures

Efficacy metrics were clinical remission (Mayo score ≤ 2, no single subscore > 1), clinical response (Mayo score ≥ 3 points lower and ≥ 30% lower than baseline, rectal bleeding subscore ≥ 1 point or ≤ 1), endoscopic remission (Mayo score ≥ 0 or 1), and mucosal healing (Mayo score ≥ 0 or 1). The safety outcome was the number of patients with any adverse events (AEs), recurrence of ulcerative colitis, infections, discontinuation due to AEs, serious AEs and serious infections.

### Study selection

The present study involved a rigorous screening process whereby identified articles were evaluated by two independent researchers (XQC and YNB) based on information presented in the title, summary, and full text. In cases of disagreement regarding the inclusion of a particular study, the third independent expert (Yaru Gu) was consulted to provide recommendations. Full-text articles were examined by both researchers to determine inclusion, and in cases where a consensus could not be reached, the third reviewer provided arbitration. Notably, multiple reports of the same study were scrutinized to ensure accurate and comprehensive assessment of relevant studies.

### Data extraction and quality assessment

In accordance with standard research practices, the present study involved careful extraction of relevant information from each individual study under consideration. Specifically, the first author, year of publication, underlying disease, patient count, study duration, demographic profile, exposure definition (including information on drug, dosage, and duration), additional adjuvant therapies, and pertinent outcomes were all meticulously identified and examined.

Moreover, consistent with our methodology, we grouped various doses of the same treatment together as being part of the same broader intervention. Should discrepancies emerge during the course of data extraction, consensus was reached by examining the relevant original records of the data.

Further, in order to ensure that the inclusion of the trials under examination was of a uniformly high caliber, we employed the Cochrane Bias Risk Tool, with both XQC and YNB serving as independent investigators.

### Statistical investigation

In this particular study, we made use of Review Manager 5.3 software to conduct a traditional meta-analysis and literature quality assessment. We employed odds ratio (OR) and 95% CI for dichotomous variables such as overall response rate, recurrence rate, and incidence of adverse reactions, while magnitude indicators for continuous variables like inflammatory factors were determined using mean difference (MD) and 95% CI. Our approach involved pairwise comparison of all included papers, revealing that no closed loops were formed in this study. The present study employed the I^2^ as the primary means to assess heterogeneity. When heterogeneity among study outcomes was found to be absent (I^2^ ≤ 50%), we used a fixed effect model to conduct meta-analysis. However, in the event of heterogeneity (I^2^ > 50%), we performed further analysis to identify the underlying sources of heterogeneity. In cases where significant clinical heterogeneity was excluded, we used the random effects model to carry out meta-analysis. The study employed a frequency-based random effects model conducted using the STATA16.0 software for network meta-analysis. The group orders of the study outcome measures were networked, and various analyses such as data processing, network evidence plots, funnel plots and area under curve (SUCRA) ranking were also conducted. The overall ranking of treatments was estimated by calculating the SUCRA for each method and using it to evaluate the benefits and harms of interventions. The magnitude of SUCRA was utilized to rank the effectiveness of interventions, where SUCRA = 1 denoted effectiveness and SUCRA = 0 indicated ineffectiveness. The publication bias of the literature was assessed using funnel plots.

## Results

### Characteristics of included studies

**Fig. 1:**
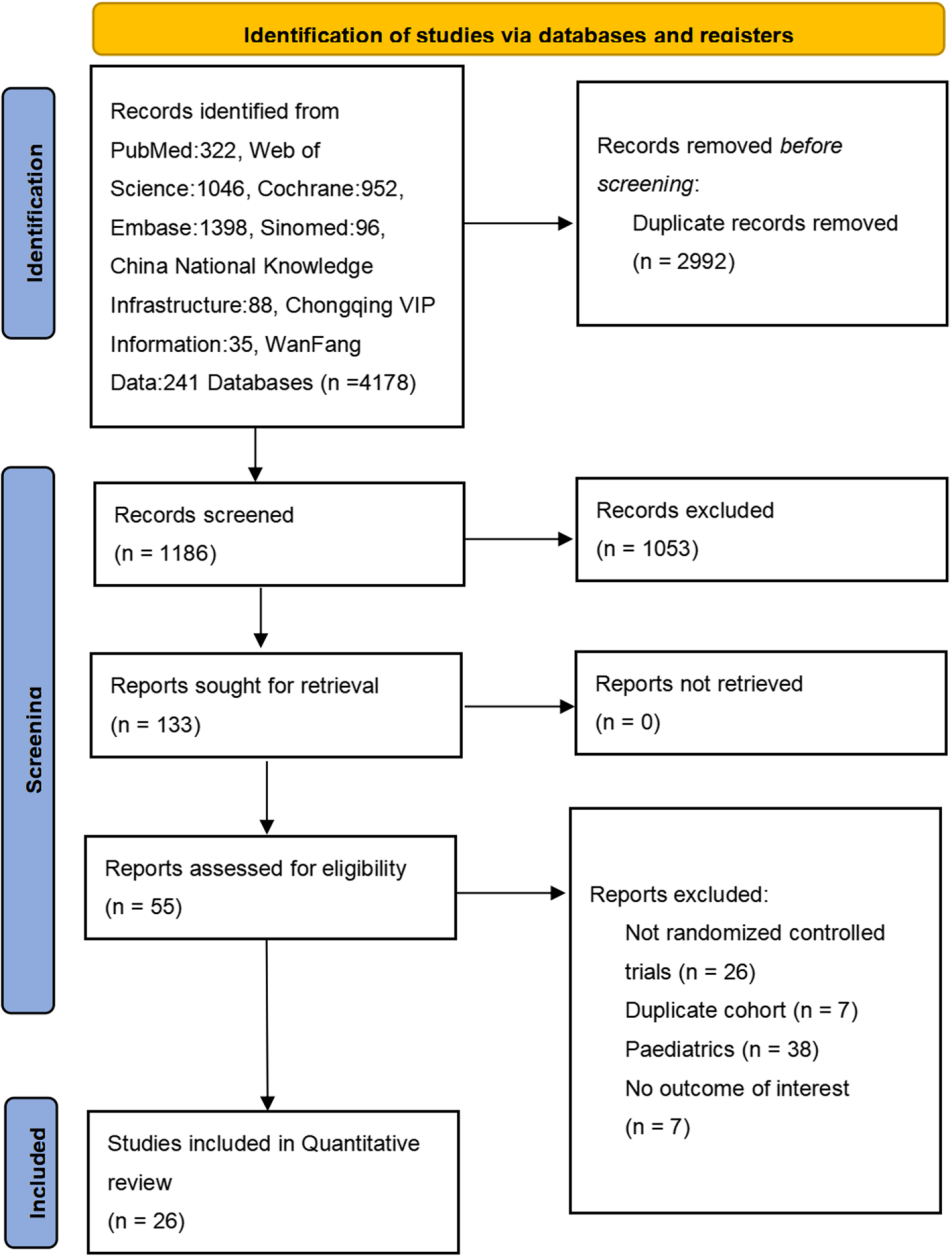
PRISMA 2020 flowchart

**Fig. 2:**
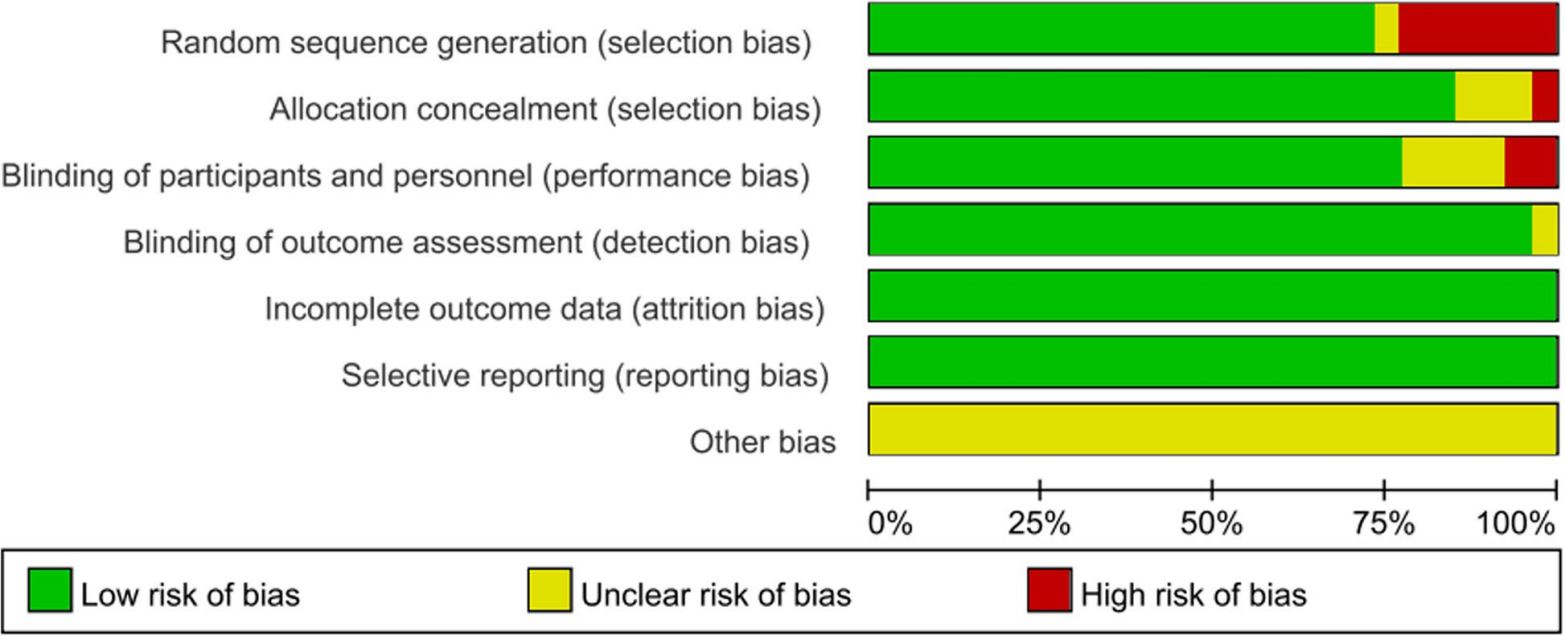
Risk of bias assessment for randomized controlled trials

The study selection process is shown in Fig. 1. A total of 4178 potentially relevant articles were initially identified through database searches, and after the exclusion of 2992 duplicates and another 1053 studies by a screening of titles and abstracts, the full texts of the remaining 55 studies were further assessed for eligibility. After full­text screening, 26 studies [14, 17–42] were included for further qualitative synthesis and met the eligibility criteria in Table 1. Twenty-one trials were placebo-controlled [19–33, 35–42], mesalazine-controlled [18], azathioprine-controlled [34], and one trial was a head-to-head RCT [20]. The characteristics of the studies included were presented. There were four of infliximab [20, 28, 41, 42], four of adalimumab [18, 20, 32, 39], three of etrolizumab [19–21], three of vedolizumab [22–24], two of ustekinumab [25, 27], one of cobitolimod [26], one of PF-00547659 [35], one of eldelumab [30], three of golimumab [31, 33, 36], one of BMS-936557 [35], one of basiliximab [38], one of visilizumab [37], and one of daclizumab [40]. Most RCTs were found to have a low or some concerns for risk of bias, and six articles were noted to have a high risk of bias in Fig. 2. There was no evidence of publication bias with funnel plot analysis in Fig. 2.

**Table 1:** Characteristics of included studies

Author (Year)	Country	phase	UC severity	concomitant drugs	Medication (Experiment)	Medication (Control)	Duration of treatment	Age (years)		Male (no./total sample size)		Weight related indicators		Duration of disease(years)	
								Experiment	Control	Experiment	Control	Experiment	Control	Experiment	Control
Brian G Feagan 2023 [17]	Canada	2	FM6-12; EMS ≥ 2	aminosalicylates and corticosteroids (prednisone or equivalent)	Guselkumab (week 0, 4, 8 200 mg)	Golimumab (0 week 200 mg, 100 mg at week2, then 100 mg every 4 week)	12 weeks	39.1(13.67)	38.1(10.47)	40/71	42/72	69.6(16.72)	73.9(17.11)	5.4(5.70)	4.7(4.48)
Wan XM 2022 [18]	China	Not reported	Not reported	-	Adamumab (0week160mg, 80 mg at week 2,then 40 mg/week)	Mesalazine	12 weeks	42. 24 (13. 46)	43. 32 (13. 21)	15/28	14/28	-	-	3.63 (2. 23)	3. 32 (2. 32)
Severine Vermeire 2022 [19]	Canada	3	FMS6-12;CRES ≥ 2; RBS ≥ 1; SFS ≥ 1	5-aminosalicylates, Oral corticosteroids, Immuno­­suppressants such as AZA, 6-mercaptopurine, and metho­trexate	Etrolizumab (105 mg once every 4 weeks)	Placebo	10 weeks	36 (18–77)*	38 (18–69)*	60/108	52/106	BMI:24 (13–80)*	BMI:25 (15–46)*	5.4 (0.6–44.0)*	5.9 (0.3–40.4)*
David T Rubin 2022 [20]	USA	3	FMS6-12; EMS ≥ 2; RBS ≥ 1	oral 5-aminosalicylates,Oral corticosteroids, Immuno–suppressants such as AZA, 6-mercaptopurine, and metho­trexate	Etrolizumab (105 mg once every 4 weeks) Adalimumab (160 mg on day 1, 80 mg at week 2;40 mg at weeks 4, 6, and 8)	Placebo	8 weeks	36.5 (18–79)* 41.0 (19–75)*	36.0 (19–78)*	74/144 82/142	39/72	BMI:22.7 (15.0–33.6)* BMI:24.3 (15.7–43.3)*	BMI:23.2 (15.6–40.6)*	3.4 (0.4–41.9)* 4.0(0.3–36.4)*	4.7 (0.3–40.8)*
					Etrolizumab (105 mg once every 4 weeks) Adalimumab (160 mg on day 1, 80 mg at week 2;40 mg at weeks 4, 6, and 8)	Placebo	8 weeks	39.0 (18–77)* 38.0 (18–71)*	36.5 (18–79)*	84/143 81/143	38/72	BMI:25.0 (16.4–48.9)* BMI:24.1 (16.4–40.7)*	BMI:23.7 (16.9–45.8)*	3.6 (0.3–58.8)* 4.1 (0.3–37.9)*	4.0 (0.3–25.4)*
Laurent Peyrin-Biroulet 2022 [21]	France	3	FMS6-12; EMS ≥ 2; RBS ≥ 1	oral 5-aminosalicylates, Oral corticosteroids, Immunosuppressants such as AZA, 6-mercaptopurine, and methotrexate	Etrolizumab (105 mg once every 4 weeks)	Placebo	14 weeks	39.0 (18–76)*	36.0 (18–76)*	224/384	54/95	BMI:24.3 (14.3–62.1)*	BMI:24.4 (15.5–46∙2)*	7.10 (0.6–44.0)*	7.36 (0.8–40.9)*
Masakazu Nagahori 2021 [22]	Japan	3	FMS6-12; EMS ≥ 2	Not reported	Vedolizumab (300 mg at Weeks 0, 2, and 6)	Placebo	6 weeks	42.3(14.4)	44.0(16.0)	99/164	55/82	-	-	7.2 (6.2)	8.6 (8.0)
Taku Kobayashi 2021 [23]	Japan	3	FM6-12; EMS ≥ 2	Oral corticosteroids or AZA or 6-mercaptopurine	Vedolizumab (108 mg Q2W) Vedolizumab (300 mg Q8W)	Placebo	44 weeks	46.0(15.5) 54.5(19.1)	43.6(13.0)	7/10 2/2	6/10	62.9(16.3) 61.8(6.8)	58.2(10.6)	8.2(8.0) 7.1(4.0)	10.3(6.6)
William J. Sandborn 2020 [24]	USA	3	FM6-12; EMS ≥ 2	Mesalamine, AZA, 6-mercaptopurine, oral corticosteroids	Vedolizumab (108 mg Q2W SC) Vedolizumab (300 mg Q8W IV)	Placebo	48 weeks	38.1 (13.1) 41.6 (14.1)	39.4 (11.7)	65/106 31/54	34/56	71.6 (17.2) 77.0 (16.9)	74.0 (20.9)	8.2 (5.9) 8.0 (6.2)	7.4 (7.1)
Remo Panaccione 2020 [25]	Canada	2A	FM6-12; EMS ≥ 2	aminosalicylic, Immunosuppressants, oral corticosteroids	Ustekinumab (90 mg Q12w) Ustekinumab (90 mg Q8w)	Placebo	44 weeks	40.4 (13.22) 39.9 (12.92)	42.9 (14.54)	77/141 82/143	73/115	71.60 (60.00; 83.80) 70.80 (59.00; 85.30)	72.00 (60.00; 79.40)	-	-
Raja Atreya 2020 [26]	Germany	2B	FM6-12; EMS ≥ 2	5-aminosalicylic acid or sulphasalazine or glucocorticosteroids	Cobitolimod (2 *31 mg) Cobitolimod (2 *125 mg) Cobitolimod (4 *125 mg) Cobitolimod (2 *250 mg)	Placebo	3 weeks	47.4 (16.4) 47.0 (16.9) 47.2 (14.9) 46.2 (14.0)	45.5 (15.2)	26/40 20/43 23/42 36/42	33/44	75.5 (16.7) 71.5 (14.9) 73.1 (17.5) 73.3 (13.2)	78∙1 (12.9)	7∙9 (6.5) 8∙5 (7.4) 8∙1 (6.8) 7∙9 (6.8)	7∙4 (7.3)
B.E. Sands 2019 [27]	USA	Not report	FM6-12; EMS ≥ 2	Aminosalicylates, corticosteroids, immunomodulators	Ustekinumab (6 mg/kg) Ustekinumab (130 mg)	Placebo	8 weeks	41.7(13.7) 42.2(13.9)	41.2(13.5)	195/320 190/322	197/319	73.0(19.3) 73.7(16.8)	72.9(16.8)	8.2(7.8) 8.1(7.2)	8.0(7.2)
Chen BL 2017 [28]	China	Not report	FMS6-12	Mercaptopurine AZA; Mesalazine Methylprednisolone	Infliximab (5 mg/kg at week 0,2,6,14,22)	Placebo	26 weeks	39.2(12.6)	37.0(11.4)	21/50	29/49	60.69(13.62)	57.16(11.63)	5.21(5.11)	5.91(5.05)
Séverine Vermeire 2017 [29]	USA	2	FMS ≥ 6; EMS ≥ 2	Not reported	PF-00547659 (7.5 mg Q4W) PF-00547659 (22.5 mg Q4W) PF-00547659 (75 mg Q4W) PF-00547659 (225 mg Q4W)	Placebo	12 weeks	41.3 (12.5) 42.1 (14.7) 37.7 (12.4) 41.3 (13.2)	38.6 (12.7)	39/71 45/70 38/73 42/70	44/73	BMI:24.3 (4.2) BMI:24.3 (4.5) BMI:25.4 (6.0) BMI:25.4 (5.8)	BMI:25.5 (6.0)	-	-
William J. Sandborn 2016 [30]	USA	2b	FMS6-12; EMS ≥ 2	Aminosalicylates, Prednisone or AZA, or 6-mercaptopurine	Eldelumab (day1-8, every other week thereafter) Eldelumab (day1-8, every other week thereafter)	Placebo	64 days	40.8 (15.1) 39.0 (12.7)	42.7 (14.2)	54/84 44/85	43/83	-	-	3.9 (0.5–39.6)* 5.0 (0.5–27.0)*	5.7 (0.5–31.2)*
P. Rutgeerts 2015 [31]	Belgium	2/3	FMS6-12; EMS ≥ 2	Not reported	golimumab (1 mg/kg) golimumab (2 mg/kg) golimumab (4 mg/kg)	Placebo	6 weeks	40.7(15.51) 42.3(13.14) 39.9(14.07)	40.9(12.58)	41/62 36/75 50/77	47/77	-	-	6.2(5.07) 7.6(8.04) 6.5(6.54)	6.8(6.59)
Yasuo Suzuki 2014 [32]	Japan	2/3	FMS6-12; EMS ≥ 2	Prednisolone, AZA, 6-mercaptopurine	Adalimumab (80 mg at week 0, then 40 mg every other week) Adalimumab (160/80 mg at weeks 0/2, then 40 mg every other week)	Placebo	52 weeks	44.4(15.0) 42.5(14.6)	41.3(13.6)	50/87 61/90	70/96	58.7(11.1) 60.1(12.3)	60.8(14.1)	8.3 (7.7) 7.8 (7.1)	7.8 (6.6)
William J. Sandborn 2014 [33]	USA	2/3	FMS6-12; EMS ≥ 2	Aminosalicylate corticosteroids	Golimumab (200 mg and then 100 mg) Golimumab (400 mg and then 200 mg)	Placebo	6 weeks	40.0(3.54) 40.7(13.75)	39.0(13.04)	180/331 201/331	175/331	-	-	6.4(6.17) 6.4(6.27)	6.0(6.65)
Remo Panaccione 2014 [34]	UK	Not reported	FMS6-12;	none	Infliximab (5 mg/kg at weeks 0,2,6) Azathioprine (2.5 mg/kg)	Infliximab + Azathioprine	16 weeks	38.5 (12.7) 40.7 (13.2)	38.0 (12.2)	42/78 33/79	48/80	-	-	6.3 (6.5) 6.6 (7.8)	5.2 (5.1)
Lloyd Mayer 2014 [35]	USA	2	FMS6-12; EMS ≥ 2	5-Aminosalicylates, prednisolone ≤ 20 mg/day,6-mercaptopurine	BMS-936557 (10 mg/kg every other week)	Placebo	8 weeks	44.7 (12.8)	41.8 (14.2)	37/55	31/54	81.9 (16.1)	74.5 (18.1)	6.7 (7.8)	5.5 (4.4)
William J. Sandborn 2014 [36]	USA	2/3	FMS6-12; EMS ≥ 2	Aminosalicylates, Corticosteroids, mesalamine	Golimumab (50 mg Q4W) Golimumab (100 mg Q4W)	Placebo	54 weeks	41.4(13.84) 39.1(13.11)	40.2 ± 14.0	77/154 89/154	75/156	-	-	6.8(6.93) 7.2(7.04)	6.9(6.96)
William J. Sandborn 2014 [37]	USA	Not reported	FMS ≥ 10; SFS ≥ 2	mesalamine, AZA, 6-mercaptopurine, or methotrexate	Visilizumab (5 mg/kg on days 1 and 2)	Placebo	45 days	40.4 (12.9)	40.8 (13.5)	52/84	28/43	75.5 (17.3)	74.8 (16.8)	6.4 (6.8)	6.8 (7.1)
BRUCE E. SANDS 2012 [38]	USA	2	FMS6-12;	Prednisone, AZA, mercaptopurine, 5-aminosalicylate	Basiliximab (20 mg, at week0,2,4) Basiliximab (40 mg, at week0,2,4)	Placebo	8 weeks	43 (14) 39 (12)	38 (11)	27/46 31/52	31/51	70.3 (15) 70.3 (19)	69.7 (18)	-	-
William J. Sandborn 2012 [39]	USA	3	FMS6-12; EMS ≥ 2	Prednisone, AZA, 6-mercaptopurine, 5-aminosalicylate	Adalimumab (160 mg at week 0, 80 mg at week 2, then 40 mg every other week)	Placebo	52 weeks	39.6(12.47)	41.3 ± 13.22	142/248	152/246	75.3(17.71)	77.1(17.31)	8.1(7.09)	8.5(7.37)
G Van Assche 2006 [40]	Japan	Not reported	FMS5-10;	Oral corticosteroid 5-aminosalicylates, methylprednisolone 32 mg/day or less (or equivalent dose of other corticosteroid), AZA, and 6-mercaptopurine were permitted	Daclizumab (1 mg/kg, at weeks 0 and 4) Daclizumab (2 mg/kg,at weeks 0, 2, 4, and 6)	Placebo	6 weeks	47.4 (14.09) 42.6 (15.37)	40.7 (13.23)	29/56 25/47	33/56	76.6 (19.45) 79.8 (19.29)	80.5 (19.24)	7.8 (6.62) 8.3 (8.06)	6.8 (7.35)
Paul Rutgeerts 2005 [41]	Belgium	Not reported	FMS6-12;	corticosteroids alone or in combination with AZA or mercaptopurine	Infliximab (5 mg/ kilogram Q8w) Infliximab (10 mg/ kilogram Q8w)	Placebo	54 weeks	42.4(14.3) 41.8(14.9)	41.4(13.7)	78/121 72/122	72/121	80.0(17.8) 76.9(17.1)	76.8(16.2)	5.9(5.4) 8.4(8.1)	6.2(5.9)
				corticosteroids alone or in combination with AZA or mercaptopurine and medications containing 5-aminosalicylates	Infliximab (5 mg/ kilogram Q8w) Infliximab (10 mg/ kilogram Q8w)	Placebo	30 weeks	40.5(13.1) 40.3(13.3)	39.9(13.5)	76/121 68/120	71/123	78.4(17.8) 79.6(20.6)	76.1(17.4)	6.7(5.3) 6.5(5.8)	6.5(6.7)
C S J Probert 2003 [42]	UK	Not reported	ulcerative colitis symptom score of 6 or more	Prednisolone 30 mg	Infliximab (5 mg/ kilogram at weeks 0, 2)	Placebo	2 weeks	41 (35.5–50.5)*	40 (29–43.5)*	-	-	66 (61–78)*	72 (60–8)*	-	-

Values are given in mean and standard deviation unless otherwise stated. *—Values given in median and range, *Q2W *Quaque 2 week, *Q4W *Quaque 4 week, *Q8W *Quaque 8 week. EMS = endoscopic Mayo subscore, *FMS* Full Mayo score, *RBS* Rectal bleeding subscore, *CRES* Centrally read endoscopic score, *SFS* Stool frequency subscore

Mayo Clinic total score [MCS] of 6–12 with a centrally read endoscopic score of ≥ 2, a rectal bleeding subscore of ≥ 1, and a stool frequency subscore of ≥ 1

### NMA of the efficacy of different monoclonal antibodies in RCTs

**Fig. 3:**
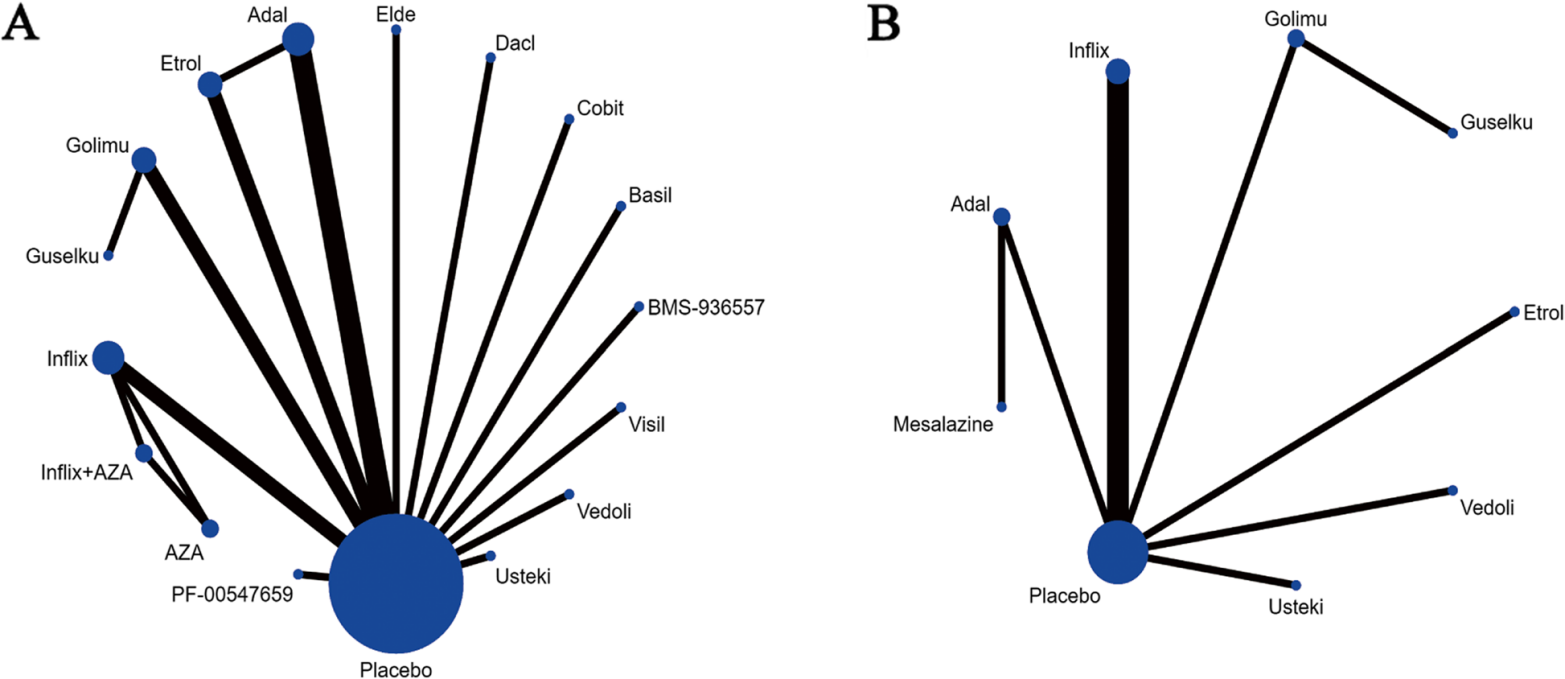
Network diagram of outcome indicators. A Induction therapy of clinical response; A Maintenance therapy of clinical response

This study involved a comparison of antibodies, including infliximab, etrolizumab, adalimumab, vedolizumab, ustekinumab, cobitolimod, PF-00547659, eldelumab, golimumab, BMS-936557, basiliximab, visilizumab, guselkumab and daclizumab. The nodes in the processing network for each antibody were sized proportionally to the number of random participants, while the thickness of each line in the network diagram was proportional to the number of lines in the network. Detailed information on this processing network is presented in Fig. 3.

### Induction therapy

Overall, 21 RCTs including patients with moderate–severe ulcerative colitis, treated with infliximab (5 trials, 1107 patients), adalimumab (2 trials, 767 patients), etrolizumab (2 trials, 1195 patients), golimumab (3 trials, 1427 patients), vedolizumab (1 trials, 246 patients), daclizumab (1 trials, 159 patients), visilizumab (1 trial, 127 patients), cobitolimod (1 trials, 211 patients), ustekinumab (1 trials, 961 patients), PF-00547659 (1 trials, 357 patients), eldelumab (1 trials, 252 patients), BMS-936557 (1 trials, 109 patients) and basiliximab (1 trials, 149 patients) were included; 1 trial compared guselkumab vs golimumab, 1 trial compared infliximab vs azathioprine, 1 trial compared etrolizumab vs adalimumab.

### Clinical remission

The assessment of biological products for their ability to induce clinical remission did not reveal any significant differences (Supplementary Table 3A). However, PF-00547659 demonstrated a significant superiority over infliximab (OR 6.36, 95%CI 1.09–37.21) and azathioprine (OR 4.22, 95% 1.93–9.22) in inducing clinical remission. According to the analysis presented in Table 2 and Supplementary Fig. 1, Vedolizumab showed the highest success rate for inducing clinical remission at 94.1%, closely followed by infliximab + azathioprine at 80.1%.

**Table 2:** SUCRA score for clinical remission, clinical response, endoscopic improve, mucosal healing, adverse events, recurrence of ulcerative colitis, infections, adverse events resulting in treatment discontinuation, serious adverse events, serious infections in induction treatments

Clinical remission		Clinical response		Endoscopic improve		Mucosal Healing		Adverse events		Recurrence of ulcerative colitis		Infections		Adverse events resulting in treatment discontinuation		serious adverse events		serious infections	
Vedoli	94.1	Vedoli	97.5	Usteki	94.4	Inflix + AZA	92.2	Cobit	88.4	Guselku	94.9	Etrol	72.5	Guselku	94.8	Guselku	78.0	Elde	65.3
Inflix + AZA	80.1	Inflix + AZA	92.7	Etrol	69.1	Inflix	91.4	Guselku	81.7	Usteki	65.6	Placebo	67.4	Golimu	77.8	Elde	75.1	Etrol	64.8
Adal	72.9	Inflix	86.9	Adal	59.6	Adal	74.1	Usteki	64.2	Adal	62.5	Guselku	64.0	Cobit	67.4	Usteki	74.4	Usteki	58.7
PF-00547659	71.7	Usteki	75	Placebo	19.4	Etrol	71.5	Etrol	61.8	Dacl	6.6	Usteki	60.4	Usteki	58.0	Golimu	66.1	Golimu	51.6
Usteki	62.6	Golimu	71.4	Cobit	7.6	AZA	64.5	Elde	53.7	Etrol	58.9	Adal	55.0	Placebo	50.7	Adal	61.3	Guselku	51.2
Etrol	60.9	PF-00547659	59.7			BMS-936557	50.3	Placebo	53.2	PF-00547659	53.1	PF-00547659	45.9	PF-00547659	47.5	Cobit	41.5	Adal	48.6
AZA	57.5	BMS-936557	56			Visil	44.2	Golimu	45.5	Golimu	48.4	Elde	42.7	Adal	34.8	Visil	39.1	Placebo	34.7
Cobit	57.4	Elde	50.3			Elde	43.4	Adal	43.7	Cobit	31.9	Golimu	25.0	Basil	27.9	Placebo	37.3	BMS-936557	25.0
Inflix	57.3	AZA	48.9			Basil	36.7	Basil	39.8	Placebo	28.1	BMS-936557	16.9	Etrol	22.6	PF-00547659	37.1		
Golimu	47.9	Etrol	45			Placebo	33.9	PF-00547659	36.2					BMS-936557	18.6	Etrol	29.7		
Elde	41.6	Adal	43.8			Golimu	26.8	BMS-936557	31.4							BMS-936557	10.4		
Guselku	34.1	Guselku	37.5			PF-00547659	15.1	Visil	0.3										
BMS-936557	28.9	Visil	35.5			Guselku	5.8												
Basil	25.4	Placebo	17.6																
Visil	24.2	Basil	15.1																
Placebo	21	Cobit	14.5																
Dacl	12.3	Dacl	2.7																

### Clinical response

All treatments except BMS-936557, azathioprine, visilizumab, basiliximab, cobitolimod and daclizumab are significantly more efficacious than placebo at inducing clinical response (Supplementary Table 3B). Among these treatments, vedolizumab has been ranked the highest (Supplementary Fig. 1, Table 2, SUCRA 97.4%), followed by infliximab + azathioprine, infliximab, and ustekinumab.

### Endoscopic improve

Ustekinumab, etrolizumab, and adalimumab are significantly more efficacious than placebo at inducing endoscopic improvement, with ustekinumab ranking highest (SUCRA 94.4%) followed by etrolizumab, adalimumab, and placebo (Supplementary Table 3C, Table 2, Supplementary Fig. 1).

### Mucosal healing

Among the 23 trials included in the analysis of mucosal healing, 18 trials were considered. The efficacy of different treatments in inducing mucosal healing was evaluated and summarized in the league table. Infliximab + azathioprine, infliximab, etrolizumab, and adalimumab were found to have statistically significant effects on the induction of mucosal healing compared to placebo (Supplementary Table 3D). According to the SUCRA table, the highest SUCRA value for mucosal healing was achieved by infliximab + azathioprine, with a value of 92.2% (Table 2, Supplementary Fig. 1).

### Maintenance therapy

A total of 13 randomized controlled trials (RCTs) were included in maintenance therapy analysis, involving patients with moderate to severe ulcerative colitis. The treatments evaluated in these trials included infliximab (1 trial, 728 patients), adalimumab (3 trials, 823 patients), golimumab (1 trial, 142 patients), vedolizumab (1 trial, 238 patients), and ustekinumab (2 trials, 922 patients). Additionally, one trial compared guselkumab to golimumab, and another trial compared adalimumab to mesalazine.

### Clinical remission

The analysis revealed moderate confidence in the estimates, indicating that vedolizumab may be more effective than ustekinumab and mesalazine in treating patients with moderate to severe ulcerative colitis (vedolizumab vs ustekinumab: OR, 3.17; 95% CI, 1.01–9.96; vedolizumab vs mesalazine: OR, 4.36; 95% CI, 1.65–11.49) (Supplementary Table 4A). In terms of maintaining clinical remission, vedolizumab (SUCRA, 89.7) and infliximab (SUCRA, 79.8) were ranked highest among the treatments evaluated in the study (Table 3, Supplementary Fig. 2).

**Table 3:** SUCRA score for clinical remission, clinical response, endoscopic improve, mucosal healing, adverse events, recurrence of ulcerative colitis, infections, adverse events resulting in treatment discontinuation, serious adverse events, serious infections in maintenance treatments

Clinical remission		Clinical response		Endoscopic improve		Mucosal Healing		Adverse events		Recurrence of ulcerative colitis		Infections		Adverse events resulting in treatment discontinuation		serious adverse events		serious infections	
Vedoli	89.7	Inflix	75.8	Vedoli	92.6	Inflix	83.6	Vedoli	78.4	Guselku	92.9	Usteki	91.3	Usteki	80.4	Usteki	75.4	Usteki	67.6
Inflix	79.8	Usteki	66	Etrol	65.2	Etrol	69.1	Etrol	73.2	Usteki	89.3	Vedoli	8.8	Guselku	71	Inflix	66.2	Adal	63.1
Adal	72.7	Golimu	59.5	Usteki	34.7	Adal	64.5	Usteki	68.1	Etrol	62.9	Placebo	77.5	Vedoli	60.1	Adal	60.3	Placebo	53.7
Golimu	55.2	Adal	57.6	Placebo	7.5	Golimu	57.6	Placebo	54.2	Inflix	50.7	Guselku	64.8	Etrol	42.8	Placebo	58.1	Etrol	53.5
Etrol	48.1	Vedoli	53.3			Guselku	12.8	Adal	35.5	Vedoli	41.9	Adal	50.8	Adal	27.9	Vedoli	52.7	Inflix	49.2
Usteki	36.2	Guselku	42.8			Placebo	12.4	Golimu	35.2	Adal	39.5	Inflix	44.6	Inflix	59.2	Guselku	34.7	Guselku	34.9
Guselku	31.5	Etrol	39.9					Inflix	33.2	Golimu	16.7	Etrol	39.7	Placebo	29.7	Etrol	29.4	Golimu	28
Mesalazine	19.5	Placebo	34.9					Guselku	22.1	Placebo	6.1	Golimu	22.5	Golimu	29	Golimu	23.3		
Placebo	17.2	Mesalazine	20.1																

### Clinical response

The assessment of biological products for their ability to maintaining clinical remission revealed that there were no discernable differences (Supplementary Table 4B). According to the rank analysis presented in Table 3 and Supplementary Fig. 2, it is more likely that infliximab (SUCRA 75.8%) outperforms other treatment regimens in terms of clinical response.

### Endoscopic improve

Vedolizumab and etrolizumab are significantly more efficacious than placebo at maintaining endoscopic improvement (vedolizumab vs placebo: OR, 4.05; 95% CI, 1.46,11.19; etrolizumab vs placebo: OR, 2.15; 95% CI, 1.01–4.54) (Supplementary Table 4C). Among these treatments, vedolizumab demonstrated the highest efficacy (SUCRA 92.6%), followed by etrolizumab, adalimumab, and ustekinumab in Table 3 and Supplementary Fig. 2.

### Mucosal healing

Nine of the 13 trials were included in the analysis of mucosal healing. Treatment efficacy for mucosal healing is shown in Supplementary Table 4D. all treatment except guselkumab are significantly more efficacious than placebo at the end of maintenance. The highest SUCRA value calculated based on mucosal healing was achieved by infliximab (83.6%) in Table 3 and Supplementary Fig. 2.

### NMA of the safety of different biologics in RCTs

The induction network for safety events (eg, all AEs, recurrence of ulcerative colitis, discontinuation due to AEs, serious AEs, and serious infections) includes 12 treatments (all treatments for discontinuation due to AEs), 14 studies (10 for discontinuation due to AEs) and 5577 patients (4391 for discontinuation due to AEs). The maintenance network includes 8 treatments (7 treatment for serious infections), 13 studies (7 serious infections), 3819 patients (299 for discontinuation due to AEs and 62 for serious infections).

Between induction treatments including placebo, a handful of significant differences in the safety events assessed are observed. For all AEs, cobitolimod is ranked highest while visilizumab is ranked lowest( Supplementary Table 5A, Table 2, Supplementary Fig. 1). For recurrence of ulcerative colitis, guselkumab is ranked highest and has significantly lower than golimumab, cobitolimod and placebo, while placebo is ranked lowest (Supplementary Table 5B, Table 2). For infections, etrolizumab and BMS-936557 are ranked highest and lowest, respectively, wtih no signigicant difference observed (Supplementary Table 5C, Table 2, Supplementary Fig. 1). For discontinuation due to AEs, guselkumab is ranked highest, while BMS-936557 is lowest, respectively, with no significant difference observed (Supplementary Table 5D, Table 2, Supplementary Fig. 1). For serious AEs, guselkumab and BMS-936557 are ranked highest and lowest, respectively, with no significant difference observed (Supplementary Table 5E, Table 2, Supplementary Fig. 1). Likewise, for serious infextions, eldelumab and BMS-936557 ranked highest and lowest, respectively, with no significant difference observed (Supplementary Table 5F, Table 2, Supplementary Fig. 1).

Between maintenance treatments including placebo, some significant differences in the safety events assessed are likewise observed. In all AEs, vedolizumab is ranked highest, while guselkumab is ranked lowest, respectively, with no significant difference observed (Supplementary Table 6A, Table 3, Supplementary Fig. 2). For recurrence of ulcerative colitis, guselkumab is ranked highest, while placebo is ranked lowest, all treatment except vedolizumab, adalimumab and golimumab have significantly lower odds than placebo (Supplementary Table 6B, Table 3). For infections, ustekinumab and golimumab are ranked highest and lowest, with no significant difference observed (Supplementary Table 6C, Table 3, Supplementary Fig. 2). For discontinuation due to AEs, ustekinumab, guselkumab vedolizumab and etrolizumab ranked first to fourth, respectively, guselkumab has significantly lower odds than infliximab (Supplementary Table 6D, Table 3, Supplementary Fig. 2). For serious AEs, ustekinumab and golimumab are ranked highest and lowest, with no significant difference observed (Supplementary Table 6E, Table 3, Supplementary Fig. 2). For serious infections, ustekinumab and golimumab are ranked highest and lowest, with no significant difference observed (Supplementary Table 6F, Table 3, Supplementary Fig. 2).

## Discussion

The primary objective of this study was to provide a comprehensive analysis of the efficacy and safety of biologics for the induction and maintenance treatment of ulcerative colitis, as evidenced by randomized controlled trials (RCTs). The study reveals that vedolizumab exhibits potential superiority over other drug regimens in inducing and maintaining clinical remission and reponse. Additionally, vedolizumab is a selective antibody to intestinal adhesion molecule-1 (α4β7) that blocks the adhesion and migration of leukocytes to the intestinal mucosa by binding to the α4β7 integrin [43]. Infliximab exhibits potential superiority over other drug regimens in maintaining clinical response. Additionally, infliximab, an anti-TNF-α antibody, was found to be remarkably effective in producing clinical response. Regarding safety, the present network meta observed a handful of significant differences between treatment and placebo for the 6 safety events assessed (all AEs, recurrence of ulcerative colitis, infections, discontinuation due to AEs, serious AEs, serious infections) during induction and maintenance. Discontinuation rates are important to consider because they may signal a balance between drug efficacy and drug safety. Specifically, guselkumab was significantly better at avoiding discontinuation due to AEs during induction and maintenance. Ustekinumab receive high SUCRA scores for serious AEs in maintenance. Ustekinumab and guselkumab are considered the more dependable and safe option for patients in terms of adverse and serious events. Through the use of SUCRA rankings, the findings contribute to the understanding of the comparative safety of various drug treatments for gastroenterological conditions, providing valuable insights for healthcare professionals in clinical practice.

This particular study is a systematic review of RCTs involving various drugs such as infliximab, etrolizumab, adalimumab, vedolizumab, ustekinumab, cobitolimod, PF-00547659, eldelumab, golimumab, BMS-936557, basiliximab, visilizumab, and daclizumab for the treatment of ulcerative colitis. To offer a detailed summary of the efficacy and safety of each therapy, the study relied on evidence extracted from 26 RCTs in relation to inducing and maintaining clinical remission, endoscopic improvement, and safety outcomes. Furthermore, the study compares these therapies indirectly to draw useful insights for clinical settings where the availability of multiple therapeutic options is increasingly common necessitating frequent updates of indirect comparisons. We have summarized the clinical characteristics of the biological preparations involved in this study and provided reference for clinicians (Supplementary Table 7).

In the past two decades, numerous biologics have transformed the management of UC, alleviating symptoms and improving mucosal healing, clinical response, and corticosteroid-free remission, which ultimately enhances quality of life. Among these biologics, Ustekinumab, a monoclonal antibody against interleukin-12 (IL-12) and interleukin-23 (IL-23), reduces intestinal inflammation by inhibiting the IL-12 and IL-23 signaling pathways, inhibiting the differentiation and activation of inflammatory cells, and reducing circulating Th17 cells in the body [44]. The efficacy of Ustekinumab for UC was tested in the Phase III UNIFI programme, consisting of a double-blind, randomised, placebo-controlled 8-week induction phase followed by a 44-week maintenance phase study. During the induction period, clinical remission rates of 130 mg (15.5%) and 6 mg/kg (15.6%) were noteworthy. at week 152, 54.1% and 56.3% of patients were in symptomatic remission in the ustekinumab q12w and q8w groups, respectively. Pharmacokinetic analysis demonstrated that serum ustekinumab concentrations (SUCs) were proportional to dose and were not affected by concomitant immunomodulatory agents or prior exposure to biologic therapy [17]. RCTs and real-world studies with ustekinumab found Ustekinumab could potentially exhibit several advantages over other competitors in UC (anti-TNF-α drugs, vedolizumab, and tofacitinib), including a favorable profile of safety, effectiveness on certain extraintestinal manifestations, and a convenient administration mode [45]. These results suggest that Ustekinumab may be a safe and efficacious therapeutic agent for UC treatment. Approved for the treatment of moderate-to-severe active UC, adalimumab, golimumab, and infliximab are valuable anti-tumour necrosis factors. In a meta-analysis conducted by Kristian Thorlund through mesh, statistical analysis revealed infliximab to be superior to adalimumab with respect to treatment outcomes [8]. On the other hand, vedolizumab is a humanized monoclonal antibody that aims to mitigate lymphocyte transit to the intestinal tract. This inhibition is achieved specifically by targeting α4-β7 heterodimer, which is expressed on the surface of intestinal-specific lymphocytes. This mechanism of action is akin to that of PF-00547659. These insights shed light on the potential of novel therapies to improve the treatment of gastrointestinal diseases. In future studies, researchers should further investigate the efficacy and safety of these promising new drugs [43]. Our study indicates that vedolizumab does not significantly impede lymphocyte transit to the brain, in contrast to other monoclonal antibodies. These findings contribute to a better understanding of the potential neurologic effects of vedolizumab treatment, which can inform clinical decision-making for patients with gastrointestinal diseases requiring immunomodulatory therapy. Future research endeavors should expand upon our work by addressing the underlying mechanisms responsible for this phenomenon, and by evaluating other potential side-effects of vedolizumab that may impact neurological or other bodily functions [18]. In a multicenter Phase 3b trial, double-blind, double-dummy, randomized, active-controlled study, the clinical efficacy of vedolizumab and adalimumab in adult patients with moderate to severe active ulcerative colitis was investigated. The study findings ultimately revealed that vedolizumab showed superior clinical remission and endoscopic improvement compared to adalimumab, but not in terms of clinical remission without the use of corticosteroids [46].

Several studies have suggested that UC patients who exhibit suboptimal response to infliximab may experience improved outcomes and heightened survival via treatment with vedolizumab. These findings suggest that vedolizumab represents a viable alternative to infliximab with comparable safety characterizations. Such results underscore the clinical potential of vedolizumab for colorectal pathologies of inflammatory origin, while also emphasizing the importance of continued research efforts to refine and optimize immunosuppressive therapies for this patient population. Further research initiatives should seek to elucidate the distinct mechanisms by which vedolizumab may confer superior treatment outcomes in comparison to infliximab for certain UC patients [47]. In their recent online meta-analysis, Welty et al. evaluated the comparative effectiveness of different therapies for the treatment of digestive disorders such as ulcerative colitis (UC). Our study compared the clinical remission, clinical response, and mucosal healing SUCRA scores, and found that vedolizumab and infliximab ranked highly and showed comparable efficacy. In a retrospective real-world single-center study conducted on biologic-naïve outpatients with moderate-to-severe UC or mild UC, vedolizumab was shown to have higher clinical response rates, better medication persistence, and a higher likelihood of achieving steroid-free remission compared to infliximab during both the induction and follow-up periods. However, the occurrence of adverse events and rates of C-reactive protein normalization were similar between the two drugs [48]. Therefore, further head-to-head trials are necessary in order to accurately assess and compare the efficacy and safety of vedolizumab and infliximab. The study revealed that ustekinumab demonstrated superior efficacy in achieving clinical response, clinical remission, and histological improvement when compared to TNF-α inhibitors, vedolizumab, and tofacitinib. Of note, the analysis also showed that ustekinumab was second in clinical response. The data suggests the potential of ustekinumab as a primary therapy option for managing UC, and further highlights the need for continued research to optimize treatment approaches for improved patient outcomes [49].

When considering the clinical use of BMS-936557, it is crucial to address the management of any adverse reactions that may arise. BMS-936557, on the other hand, directly prohibits IP-10-related intestinal epithelial cell dysfunction, thus raising barrier integrity. Additionally, it exhibits an adequate level of tolerance, but its safety evaluation was mediocre. Notably, an association between higher drug exposure and improved clinical response and histological advancement has been identified. Further research is needed to fully elucidate these therapeutic agents' efficacy and potential benefits to the clinical management of relevant conditions [35].

However, there were some limitations to this study. Foremost, variations existed in the studies included in the analysis, primarily due to inconsistencies in the assessment of endoscopy results, mainly in earlier trials. Although most studies had assessed adverse reactions and read endoscopy results, discrepancies in their evaluation arose. Secondly, this study divulges limitations in fully examining the efficacy of biologics, given the exclusion of additional evaluative indicators such as biochemical and quality of life measures. Furthermore, the examination of small-molecule drugs such as upadacitinib remains unexplored and requires further exploration in future research. We were unable to perform subgroup analyses of Bio-exposed Populations and Bio-naive Populations as the inclusion literature all required that patients had not been previously treated with other biological agents. This investigation solely analyzed clinical trial data on previously published biologics; thus, meeting summaries, letters, and other related publications were not included. To enhance the analysis, it is vital to include the Phase 3 clinical findings of novel biologics in future work for updated analysis.

## Conclusions

This network meta-analysis (NMA) investigated the effectiveness of 13 biologics employed as induction therapy in addressing ulcerative colitis. The findings of the study indicated that infliximab and vedolizumab exhibit considerable clinical efficacy. Ustekinumab appears to have a favorable safety and effective profile during inducing and maintaining. In contrast, BMS-936557 exhibited high incidences of adverse reactions, necessitating caution when using the biologic. These findings can be beneficial to clinicians seeking to select the optimal biologic for treating ulcerative colitis, particularly as the number of therapeutic options for treating the condition increases. Furthermore, the study's results could inform forthcoming guidelines on biologic ulcerative colitis treatment. However, the analysis's reliability necessitates additional clinical practice comparisons, real-world validation, and long-term research to establish the biologics' safety and efficacy.

### Supplementary Information

**Additional file 1. **

## Data Availability

All data generated or analysed during this study are included in this published article and its supplementary information files.

## Abbreviations

AEs: Adverse events
Adal: Adalimumab
AZA: Azathioprine
Basil: Basiliximab
Cobit: Cobitolimod
CRES: Centrally read endoscopic score
Dacl: Daclizumab
Elde: Eldelumab
EMS: Endoscopic Mayo subscore
FMS: Full Mayo score
Golimu: Golimumab
Guselku: Guselkumab
MD: Mean difference
NMA: Network meta-analysis
OR: Odds ratio
PRISMANMA: Preferred Reporting Items for Systematic Reviews and Meta-Analyses for Network Meta-Analyses
Q2W: Quaque 2 week
Q4W: Quaque 4 week
Q8W: Quaque 8 week
RCTs: Randomised controlled trials
RBS: Rectal bleeding subscore
SAEs: Severe adverse events
SUCRA: Surface under cumulative ranking
SFS: Stool frequency subscore
Vedoli: Vedolizumab
Visil: Visilizumab
UC: Ulcerative colitis
Usteki: Ustekinumab
