# Supplementary material for: Network meta-analysis on efficacy and safety of different biologics for ulcerative colitis
Source: BMC Gastroenterol. 2023 Oct 6;23:346. doi: 10.1186/s12876-023-02938-6 (PMC10557260; doi:10.1186/s12876-023-02938-6)
Supplement: Supplementary file 1 — Additional file 1. [file 12876_2023_2938_MOESM1_ESM.docx]

**Supplementary Table 1.** PRISMA NMA Checklist of Items to Include When Reporting A Systematic Review Involving a Network Meta-analysis

| **Section/Topic** | **Item #** | **Checklist Item** | **Reported on Page #** |
| --- | --- | --- | --- |
| **TITLE** |  |  |  |
| Title | 1 | Identify the report as a systematic review *incorporating a network meta-analysis (or related form of meta-analysis).* | ***1*** |
|  |  |  |  |
| **ABSTRACT** |  |  |  |
| Structured summary | 2 | Provide a structured summary including, as applicable:  **Background:** main objectives  **Methods:** data sources; study eligibility criteria, participants, and interventions; study appraisal; and *synthesis methods, such as network meta-analysis.*  **Results:** number of studies and participants identified; summary estimates with corresponding confidence/credible intervals; *treatment rankings may also be discussed. Authors may choose to summarize pairwise comparisons against a chosen treatment included in their analyses for brevity.*  **Discussion/Conclusions:** limitations; conclusions and implications of findings.  **Other:** primary source of funding; systematic review registration number with registry name. | *2-4* |
|  |  |  |  |
| **INTRODUCTION** |  |  |  |
| Rationale | 3 | Describe the rationale for the review in the context of what is already known*, including mention of why a network meta-analysis has been conducted.* | ***6*** |
| Objectives | 4 | Provide an explicit statement of questions being addressed, with reference to participants, interventions, comparisons, outcomes, and study design (PICOS). | 6 |
|  |  |  |  |
| **METHODS** |  |  |  |
| Protocol and registration | 5 | Indicate whether a review protocol exists and if and where it can be accessed (e.g., Web address); and, if available, provide registration information, including registration number. | 6 |
| Eligibility criteria | 6 | Specify study characteristics (e.g., PICOS, length of follow-up) and report characteristics (e.g., years considered, language, publication status) used as criteria for eligibility, giving rationale. *Clearly describe eligible treatments included in the treatment network, and note whether any have been clustered or merged into the same node (with justification).* | ***7*** |
| Information sources | 7 | Describe all information sources (e.g., databases with dates of coverage, contact with study authors to identify additional studies) in the search and date last searched. | 6-7 |
| Search | 8 | Present full electronic search strategy for at least one database, including any limits used, such that it could be repeated. | 7, ***Supplementary Table 2*** |
| Study selection | 9 | State the process for selecting studies (i.e., screening, eligibility, included in systematic review, and, if applicable, included in the meta-analysis). | 7 |
| Data collection process | 10 | Describe method of data extraction from reports (e.g., piloted forms, independently, in duplicate) and any processes for obtaining and confirming data from investigators. | 7-8 |
| Data items | 11 | List and define all variables for which data were sought (e.g., PICOS, funding sources) and any assumptions and simplifications made. | 7-8 |
| **Geometry of the network** | **S1** | Describe methods used to explore the geometry of the treatment network under study and potential biases related to it. This should include how the evidence base has been graphically summarized for presentation, and what characteristics were compiled and used to describe the evidence base to readers. | ***8-9*** |
| Risk of bias within individual studies | 12 | Describe methods used for assessing risk of bias of individual studies (including specification of whether this was done at the study or outcome level), and how this information is to be used in any data synthesis. | 8-9 |
| Summary measures | 13 | State the principal summary measures (e.g., risk ratio, difference in means). *Also describe the use of additional summary measures assessed, such as treatment rankings and surface under the cumulative ranking curve (SUCRA) values, as well as modified approaches used to present summary findings from meta-analyses.* | 9-10 |
| Planned methods of analysis | 14 | Describe the methods of handling data and combining results of studies for each network meta-analysis. This should include, but not be limited to:   - *Handling of multi-arm trials;* - *Selection of variance structure;* - *Selection of prior distributions in Bayesian analyses; and* - *Assessment of model fit.* | 9-10 |
| **Assessment of Inconsistency** | **S2** | Describe the statistical methods used to evaluate the agreement of direct and indirect evidence in the treatment network(s) studied. Describe efforts taken to address its presence when found. | 9-10 |
| Risk of bias across studies | 15 | Specify any assessment of risk of bias that may affect the cumulative evidence (e.g., publication bias, selective reporting within studies). | **11** |
| Additional analyses | 16 | Describe methods of additional analyses if done, indicating which were pre-specified. This may include, but not be limited to, the following:   - Sensitivity or subgroup analyses; - Meta-regression analyses; - *Alternative formulations of the treatment network; and* - *Use of alternative prior distributions for Bayesian analyses (if applicable).* | 9-10 |
|  |  |  |  |
| **RESULTS†** |  |  |  |
| Study selection | 17 | Give numbers of studies screened, assessed for eligibility, and included in the review, with reasons for exclusions at each stage, ideally with a flow diagram. | Figure 1 |
| **Presentation of network structure** | **S3** | Provide a network graph of the included studies to enable visualization of the geometry of the treatment network. | Figure 3 |
| **Summary of network geometry** | **S4** | Provide a brief overview of characteristics of the treatment network. This may include commentary on the abundance of trials and randomized patients for the different interventions and pairwise comparisons in the network, gaps of evidence in the treatment network, and potential biases reflected by the network structure. | ***11*** |
| Study characteristics | 18 | For each study, present characteristics for which data were extracted (e.g., study size, PICOS, follow-up period) and provide the citations. | Table 1 |
| Risk of bias within studies | 19 | Present data on risk of bias of each study and, if available, any outcome level assessment. | Figure 2 |
| Results of individual studies | 20 | For all outcomes considered (benefits or harms), present, for each study: 1) simple summary data for each intervention group, and 2) effect estimates and confidence intervals. *Modified approaches may be needed to deal with information from larger networks.* | ***11-18*** |
| Synthesis of results | 21 | Present results of each meta-analysis done, including confidence/credible intervals. *In larger networks, authors may focus on comparisons versus a particular comparator (e.g. placebo or standard care), with full findings presented in an appendix. League tables and forest plots may be considered to summarize pairwise comparisons.* If additional summary measures were explored (such as treatment rankings), these should also be presented. | ***11-18*** |
| **Exploration for inconsistency** | **S5** | Describe results from investigations of inconsistency. This may include such information as measures of model fit to compare consistency and inconsistency models, *P* values from statistical tests, or summary of inconsistency estimates from different parts of the treatment network. | ***11-18*** |
| Risk of bias across studies | 22 | Present results of any assessment of risk of bias across studies for the evidence base being studied. | ***11-18*** |
| Results of additional analyses | 23 | Give results of additional analyses, if done (e.g., sensitivity or subgroup analyses, meta-regression analyses*, alternative network geometries studied, alternative choice of prior distributions for Bayesian analyses,* and so forth). | ***11-18*** |
|  |  |  |  |
| **DISCUSSION** |  |  |  |
| Summary of evidence | 24 | Summarize the main findings, including the strength of evidence for each main outcome; consider their relevance to key groups (e.g., healthcare providers, users, and policy-makers). | 18-19 |
| Limitations | 25 | Discuss limitations at study and outcome level (e.g., risk of bias), and at review level (e.g., incomplete retrieval of identified research, reporting bias). *Comment on the validity of the assumptions, such as transitivity and consistency. Comment on any concerns regarding network geometry (e.g., avoidance of certain comparisons).* | 23-24 |
| Conclusions | 26 | Provide a general interpretation of the results in the context of other evidence, and implications for future research. | 24 |
|  |  |  |  |
| **FUNDING** |  |  |  |
| Funding | 27 | Describe sources of funding for the systematic review and other support (e.g., supply of data); role of funders for the systematic review. This should also include information regarding whether funding has been received from manufacturers of treatments in the network and/or whether some of the authors are content experts with professional conflicts of interest that could affect use of treatments in the network. | ***26-27*** |

**Supplementary Table 2.** Search strategy

**Search Strategy in PUBMED**

((("Colitis, Ulcerative"[Mesh]) OR ((((Idiopathic Proctocolitis[Title/Abstract]) OR (Ulcerative Colitis[Title/Abstract])) OR (Colitis Gravis[Title/Abstract])) OR (Inflammatory Bowel Disease, Ulcerative Colitis Type[Title/Abstract]))) AND ((((((((((((((("Basiliximab"[Mesh]) OR (((Simulect[Title/Abstract]) OR (CHI 621[Title/Abstract])) OR (SDZ CHI 621[Title/Abstract]))) OR ("Basiliximab"[Mesh])) OR ("BMS-936557" [Supplementary Concept])) OR (("ontamalimab" [Supplementary Concept]) OR (((SHP-647[Title/Abstract]) OR (SHP647[Title/Abstract])) OR (PF-00547659[Title/Abstract])))) OR ("cobitolimod" [Supplementary Concept])) OR (("Daclizumab"[Mesh]) OR (((((((Dacliximab[Title/Abstract]) OR (Zinbryta[Title/Abstract])) OR (Zenapax[Title/Abstract])) OR (Ro 24-7375[Title/Abstract])) OR (Ro 24 7375[Title/Abstract])) OR (Ro 247375[Title/Abstract])) OR (Ro-24-7375[Title/Abstract])))) OR (("visilizumab" [Supplementary Concept]) OR ((((HuM291[Title/Abstract]) OR (HUM-291[Title/Abstract])) OR (SMART anti-CD3[Title/Abstract])) OR (Nuvion[Title/Abstract])))) OR (("golimumab" [Supplementary Concept]) OR ((((CNTO-148[Title/Abstract]) ) OR (CNTO 148[Title/Abstract])) OR (Simponi[Title/Abstract])))) OR ("eldelumab" [Supplementary Concept])) OR (("Ustekinumab"[Mesh]) OR (((Stelara[Title/Abstract]) OR (CNTO 1275[Title/Abstract])) OR (CNTO-1275[Title/Abstract])))) OR (("vedolizumab" [Supplementary Concept]) OR (((((Entyvio[Title/Abstract]) OR (MLN0002[Title/Abstract])) OR (MLN02[Title/Abstract])) OR (MLN-0002[Title/Abstract])) OR (MLN-02[Title/Abstract])))) OR (("Adalimumab"[Mesh]) OR (((((((Humira[Title/Abstract]) OR (Adalimumab-adbm[Title/Abstract])) OR (Amjevita[Title/Abstract])) OR (Adalimumab-atto[Title/Abstract])) OR (Cyltezo[Title/Abstract])) OR (D2E7 Antibody[Title/Abstract])) OR (Antibody, D2E7[Title/Abstract])))) OR (((((((rhuMAb Beta7[Title/Abstract]) OR (ANTI-BETA7[Title/Abstract])) OR (ANTI-.BETA.7[Title/Abstract])) OR (RHUMAB .BETA.7[Title/Abstract])) OR (PRO145223[Title/Abstract])) OR (PRO-145223[Title/Abstract])) OR ("etrolizumab" [Supplementary Concept]))) OR ((((((((((((MAb cA2[Title/Abstract]) OR (Monoclonal Antibody cA2[Title/Abstract])) OR (Antibody cA2, Monoclonal[Title/Abstract])) OR (cA2, Monoclonal Antibody[Title/Abstract])) OR (Infliximab-dyyb[Title/Abstract])) OR (Infliximab dyyb[Title/Abstract])) OR (Inflectra[Title/Abstract])) OR (Remicade[Title/Abstract])) OR (Infliximab-abda[Title/Abstract])) OR (Infliximab abda[Title/Abstract])) OR (Renflexis[Title/Abstract])) OR ("Infliximab"[Mesh])))) AND (randomized controlled trial[Publication Type] OR randomized[Title/Abstract] OR placebo[Title/Abstract])

**322 Results**

**Search Strategy in Embase**

| Number | String | Results |
| --- | --- | --- |
| #41 | #5 AND #39 AND #40 | 1398 |
| #40 | 'randomized controlled trial':ab,ti OR 'randomized':ab,ti OR 'placebo':ab,ti OR 'rct':ab,ti | 1145606 |
| #39 | #8 OR #11 OR #14 OR #17 OR #20 OR #21 OR #24 OR #25 OR #28 OR #29 OR #33 OR #35 OR #38 | 93862 |
| #38 | #36 OR #37 | 6489 |
| #37 | 'dacliximab':ab,ti OR 'zinbryta':ab,ti OR 'zenapax':ab,ti OR 'ro 24-7375':ab,ti OR 'ro 24 7375':ab,ti OR 'ro 247375':ab,ti OR 'ro-24-7375':ab,ti | 111 |
| #36 | 'daclizumab'/exp | 6465 |
| #35 | #30 AND #34 | 255 |
| #34 | 'basiliximab'/exp | 13398 |
| #33 | #31 OR #32 | 336 |
| #32 | 'hum291':ab,ti OR 'hum-291':ab,ti OR 'smart anti-cd3':ab,ti OR 'nuvion':ab,ti | 16 |
| #31 | 'visilizumab'/exp | 332 |
| #30 | 'simulect':ab,ti OR 'chi 621':ab,ti OR 'sdz chi 621':ab,ti | 294 |
| #29 | 'bms-936557'/exp | 71 |
| #28 | #26 OR #27 | 9855 |
| #27 | 'cnto-148':ab,ti OR 'cnto 148':ab,ti OR 'simponi':ab,ti | 72 |
| #26 | 'golimumab'/exp | 9853 |
| #25 | 'eldelumab'/exp | 71 |
| #24 | #22 OR #23 | 186 |
| #23 | 'shp-647':ab,ti OR 'shp647':ab,ti OR 'pf-00547659':ab,ti | 69 |
| #22 | 'pf-00547659'/exp | 156 |
| #21 | 'cobitolimod'/exp | 46 |
| #20 | #18 OR #19 | 12431 |
| #19 | 'stelara':ab,ti OR 'cnto 1275':ab,ti OR 'cnto-1275':ab,ti | 105 |
| #18 | 'ustekinumab'/exp | 12427 |
| #17 | #15 OR #16 | 7794 |
| #16 | 'entyvio':ab,ti OR 'mln0002':ab,ti OR 'mln02':ab,ti OR 'mln-0002':ab,ti OR 'mln-02':ab,ti | 96 |
| #15 | 'vedolizumab'/exp | 7787 |
| #14 | #12 OR #13 | 44692 |
| #13 | 'humira':ab,ti OR 'adalimumab-adbm':ab,ti OR 'amjevita':ab,ti OR 'adalimumab-atto':ab,ti OR 'cyltezo':ab,ti OR 'd2e7 antibody':ab,ti OR 'antibody, d2e7':ab,ti | 832 |
| #12 | 'adalimumab'/exp | 44643 |
| #11 | #9 OR #10 | 422 |
| #10 | 'rhumab beta7':ab,ti OR 'anti-beta7':ab,ti OR 'anti-.beta.7':ab,ti OR 'rhumab .beta.7':ab,ti OR 'pro145223':ab,ti OR 'pro-145223':ab,ti | 14 |
| #9 | 'etrolizumab'/exp | 414 |
| #8 | #6 OR #7 | 62062 |
| #7 | 'mab ca2':ab,ti OR 'monoclonal antibody ca2':ab,ti OR 'antibody ca2, monoclonal':ab,ti OR 'ca2, monoclonal antibody':ab,ti OR 'infliximab-dyyb':ab,ti OR 'infliximab dyyb':ab,ti OR 'inflectra':ab,ti OR 'remicade':ab,ti OR 'infliximab-abda':ab,ti OR 'infliximab abda':ab,ti OR 'renflexis':ab,ti | 1125 |
| #6 | 'infliximab'/exp | 62000 |
| #5 | #1 OR #2 OR #3 OR #4 | 96933 |
| #4 | 'inflammatory bowel disease, ulcerative colitis type':ab,ti | 1 |
| #3 | 'colitis gravis':ab,ti | 6 |
| #2 | 'idiopathic proctocolitis':ab,ti | 44 |
| #1 | 'ulcerative colitis'/exp | 96906 |

**1398 Results**

**Search Strategy in Web of science**

| Number | String | Results |
| --- | --- | --- |
| 1 | TS=(Ulcerative colitis OR Idiopathic Proctocolitis OR Ulcerative Colitis OR Colitis Gravis OR Inflammatory Bowel Disease, Ulcerative Colitis Type) | 73306 |
| 2 | TS=(Infliximab OR MAb cA2 OR Monoclonal Antibody cA2 OR Antibody cA2, Monoclonal OR cA2, Monoclonal Antibody OR Infliximab-dyyb OR Infliximab dyyb OR Inflectra OR Remicade OR Infliximab-abda OR Infliximab abda OR Renflexis) | 31720 |
| 3 | TS=(Etrolizumab OR rhuMAb Beta7 OR ANTI-BETA7 OR ANTI-.BETA.7 OR RHUMAB .BETA.7 OR PRO145223 OR  PRO-145223) | 181 |
| 4 | TS=(Adalimumab OR Humira OR Adalimumab-adbm OR Amjevita OR Adalimumab-atto OR Cyltezo OR D2E7 Antibody OR Antibody, D2E7) | 15712 |
| 5 | TS=(Vedolizumab OR Entyvio OR MLN0002 OR MLN02 OR MLN-0002 OR MLN-02) | 3246 |
| 6 | TS=(Ustekinumab OR Stelara OR CNTO 1275 OR CNTO-1275) | 4795 |
| 7 | TS=(Cobitolimod) | 15 |
| 8 | TS=(PF-00547659 OR SHP-647 OR SHP647 OR PF-00547659) | 59 |
| 9 | TS=(Eldelumab) | 7 |
| 10 | TS=(Golimumab OR CNTO-148 OR CNTO 148 OR Simponi) | 2259 |
| 11 | TS=(Basiliximab OR Simulect OR CHI 621 OR SDZ CHI 621) | 2478 |
| 12 | TS=(BMS-936557) | 6 |
| 13 | TS=(Visilizumab OR HuM291 OR HUM-291 OR SMART anti-CD3 OR Nuvion) | 78 |
| 14 | TS=(Daclizumab OR Dacliximab OR Zinbryta OR Zenapax OR Ro 24-7375 OR Ro 24 7375 OR Ro 247375 OR Ro-24-7375) | 1547 |
| 15 | #2 OR #3 OR #4 OR #5 OR #6 OR #7 OR #8 OR #9 OR #10 OR #11 OR #12 OR #13 OR #14 | 51040 |
| 16 | TS=(Randomized controlled trial OR Randomized OR Placebo OR RCT) | 1211775 |
| 17 | #1 AND #15 AND #16 | 1046 |

**1046 Results**

**Search Strategy in the Cochrane CENTRAL Library**

| Number | String | Results |
| --- | --- | --- |
| #1 | Ulcerative colitis | 6254 |
| #2 | (Idiopathic Proctocolitis):ab,ti,kw OR (Ulcerative Colitis):ab,ti,kw OR (Colitis Gravis):ab,ti,kw OR (Inflammatory Bowel Disease, Ulcerative Colitis Type):ab,ti,kw | 6150 |
| #3 | #1 OR #2 | 6269 |
| #4 | Etrolizumab | 75 |
| #5 | (rhuMAb Beta7):ab,ti,kw OR (ANTI-BETA7):ab,ti,kw OR (ANTI-.BETA.7):ab,ti,kw OR (RHUMAB .BETA.7):ab,ti,kw OR (PRO145223):ab,ti,kw OR (PRO-145223):ab,ti,kw | 8 |
| #6 | #4 OR #5 | 79 |
| #7 | infliximab | 2768 |
| #8 | (MAb cA2):ab,ti,kw OR (Monoclonal Antibody cA2):ab,ti,kw OR (Antibody cA2, Monoclonal):ab,ti,kw OR (cA2, Monoclonal Antibody):ab,ti,kw OR (Infliximab-dyyb):ab,ti,kw OR (Infliximab dyyb):ab,ti,kw OR (Inflectra):ab,ti,kw OR (Remicade):ab,ti,kw OR (Infliximab-abda):ab,ti,kw OR (Infliximab abda):ab,ti,kw OR (Renflexis):ab,ti,kw | 282 |
| #9 | #7 OR #8 | 2768 |
| #10 | Adalimumab | 3909 |
| #11 | (Humira):ab,ti,kw OR (Adalimumab-adbm):ab,ti,kw OR (Amjevita):ab,ti,kw OR (Adalimumab-atto):ab,ti,kw OR (Cyltezo):ab,ti,kw OR (D2E7 Antibody):ab,ti,kw OR (Antibody, D2E7):ab,ti,kw | 445 |
| #12 | #10 OR #11 | 3927 |
| #13 | Vedolizumab | 570 |
| #14 | (Entyvio):ab,ti,kw OR (MLN0002):ab,ti,kw OR (MLN02):ab,ti,kw OR (MLN-0002):ab,ti,kw OR (MLN-02):ab,ti,kw | 44 |
| #15 | #13 OR #14 | 576 |
| #16 | golimumab | 827 |
| #17 | (CNTO-148):ab,ti,kw OR (CNTO 148):ab,ti,kw OR (Simponi):ab,ti,kw | 65 |
| #18 | #16 OR #17 | 833 |
| #19 | Basiliximab | 973 |
| #20 | (Simulect):ab,ti,kw OR (CHI 621):ab,ti,kw OR (SDZ CHI 621):ab,ti,kw | 236 |
| #21 | #19 OR #20 | 1097 |
| #22 | Ustekinumab | 1160 |
| #23 | (Stelara):ab,ti,kw OR (CNTO 1275):ab,ti,kw OR (CNTO-1275):ab,ti,kw | 117 |
| #24 | #22 OR #23 | 1170 |
| #25 | Eldelumab | 8 |
| #26 | Visilizumab | 23 |
| #27 | (HuM291):ab,ti,kw OR (HUM-291):ab,ti,kw OR (SMART anti-CD3):ab,ti,kw OR (Nuvion):ab,ti,kw | 7 |
| #28 | #26 OR #27 | 23 |
| #29 | Daclizumab | 541 |
| #30 | Cobitolimod | 15 |
| #31 | PF-00547659 | 20 |
| #32 | (SHP-647):ab,ti,kw OR (SHP647):ab,ti,kw OR (PF-00547659):ab,ti,kw | 59 |
| #33 | #31 OR #32 | 59 |
| #34 | BMS-936557 | 9 |
| #35 | (Randomized controlled trial):ab,ti,kw OR (Randomized):ab,ti,kw OR (Placebo):ab,ti,kw OR (RCT):ab,ti,kw | 1207681 |
| #36 | #6 OR #9 OR #12 OR #15 OR #18 OR #21 OR #24 OR #25 OR #28 OR #29 OR #30 OR #33 OR #34 | 9541 |
| #37 | #3 AND #36 AND #35 | 952 |

**Supplementary Table 3A.** League table for clinica remission in induction therapy.

| Vedoli | 0.32 (0.03,3.89) | **0.07 (0.01,0.68)** | **0.06 (0.01,0.32)** | 0.21 (0.03,1.72) | **0.13 (0.02,0.89)** | **0.03 (0.00,0.35)** | 0.41 (0.04,4.46) | 0.27 (0.05,1.62) | **0.09 (0.01,0.93)** | 0.19 (0.03,1.25) | 0.18 (0.03,1.22) | **0.06 (0.01,0.56)** | 0.19 (0.02,1.99) | **0.06 (0.01,0.63)** | 0.19 (0.02,2.11) | **0.11 (0.01,0.99)** |
| --- | --- | --- | --- | --- | --- | --- | --- | --- | --- | --- | --- | --- | --- | --- | --- | --- |
|  | Inflix+AZA | 0.22 (0.02,2.67) | 0.20 (0.03,1.35) | 0.66 (0.06,6.81) | 0.42 (0.05,3.61) | 0.10 (0.01,1.34) | 1.28 (0.09,17.18) | 0.85 (0.11,6.65) | 0.27 (0.02,3.58) | 0.60 (0.07,5.10) | 0.56 (0.06,4.95) | 0.20 (0.02,2.22) | 0.58 (0.04,7.69) | 0.18 (0.01,2.43) | 0.59 (0.04,8.12) | 0.34 (0.03,3.87) |
|  |  | Adal | 0.90 (0.18,4.41) | 2.95 (0.37,23.76) | 1.87 (0.29,12.31) | 0.46 (0.04,4.80) | 5.73 (0.53,61.61) | 3.80 (0.65,22.31) | 1.19 (0.11,12.84) | 2.70 (0.42,17.29) | 2.52 (0.38,16.88) | 0.90 (0.10,7.80) | 2.58 (0.24,27.56) | 0.80 (0.07,8.73) | 2.67 (0.24,29.16) | 1.53 (0.17,13.64) |
|  |  |  | PF-00547659 | 3.28 (0.85,12.67) | 2.08 (0.76,5.71) | 0.52 (0.09,2.86) | **6.36 (1.09,37.21)** | **4.22 (1.93,9.22)** | 1.33 (0.23,7.76) | **3.00 (1.15,7.84)** | 2.80 (0.99,7.95) | 1.01 (0.23,4.31) | 2.87 (0.50,16.60) | 0.89 (0.15,5.30) | 2.97 (0.50,17.70) | 1.70 (0.38,7.64) |
|  |  |  |  | Usteki | 0.64 (0.12,3.43) | 0.16 (0.02,1.40) | 1.94 (0.21,17.95) | 1.29 (0.27,6.13) | 0.40 (0.04,3.74) | 0.92 (0.17,4.81) | 0.86 (0.16,4.72) | 0.31 (0.04,2.23) | 0.88 (0.10,8.02) | 0.27 (0.03,2.55) | 0.91 (0.10,8.50) | 0.52 (0.07,3.92) |
|  |  |  |  |  | Etrol | 0.25 (0.03,1.81) | 3.06 (0.40,23.32) | 2.02 (0.56,7.26) | 0.64 (0.15,2.72) | 1.44 (0.36,5.80) | 1.35 (0.32,5.73) | 0.48 (0.08,2.83) | 1.38 (0.18,10.43) | 0.43 (0.05,3.32) | 1.42 (0.18,11.06) | 0.82 (0.13,4.99) |
|  |  |  |  |  |  | Cobit | **12.35 (1.05,144.72)** | **8.18 (1.24,53.87)** | 2.57 (0.22,30.16) | 5.82 (0.82,41.55) | 5.44 (0.73,40.46) | 1.95 (0.21,18.48) | 5.57 (0.48,64.76) | 1.72 (0.14,20.49) | 5.76 (0.48,68.45) | 3.31 (0.34,32.26) |
|  |  |  |  |  |  |  | Inflix | 0.66 (0.10,4.57) | 0.21 (0.02,2.53) | 0.47 (0.06,3.52) | 0.44 (0.11,1.83) | 0.16 (0.02,1.56) | 0.45 (0.04,5.44) | 0.14 (0.01,1.72) | 0.47 (0.11,1.93) | 0.27 (0.03,2.72) |
|  |  |  |  |  |  |  |  | AZA | 0.31 (0.05,2.17) | 0.71 (0.25,2.03) | 0.66 (0.18,2.45) | 0.24 (0.05,1.24) | 0.68 (0.10,4.65) | 0.21 (0.03,1.48) | 0.70 (0.10,4.95) | 0.40 (0.07,2.19) |
|  |  |  |  |  |  |  |  |  | Golimu | 2.26 (0.30,16.92) | 2.12 (0.27,16.44) | 0.76 (0.08,7.48) | 2.17 (0.18,26.12) | 0.67 (0.05,8.26) | 2.24 (0.18,27.58) | 1.29 (0.13,13.05) |
|  |  |  |  |  |  |  |  |  |  | Elde | 0.93 (0.23,3.86) | 0.33 (0.06,1.91) | 0.96 (0.13,7.07) | 0.30 (0.04,2.25) | 0.99 (0.13,7.51) | 0.57 (0.10,3.37) |
|  |  |  |  |  |  |  |  |  |  |  | Guselku | 0.36 (0.06,2.15) | 1.02 (0.13,7.88) | 0.32 (0.04,2.51) | 1.06 (0.25,4.52) | 0.61 (0.10,3.77) |
|  |  |  |  |  |  |  |  |  |  |  |  | BMS-936557 | 2.86 (0.29,27.92) | 0.88 (0.09,8.85) | 2.95 (0.30,29.55) | 1.70 (0.21,13.71) |
|  |  |  |  |  |  |  |  |  |  |  |  |  | Basil | 0.31 (0.03,3.78) | 1.03 (0.08,12.63) | 0.59 (0.06,5.97) |
|  |  |  |  |  |  |  |  |  |  |  |  |  |  | Visil | 3.35 (0.27,41.93) | 1.92 (0.19,19.85) |
|  |  |  |  |  |  |  |  |  |  |  |  |  |  |  | Placebo | 0.57 (0.06,5.91) |
|  |  |  |  |  |  |  |  |  |  |  |  |  |  |  |  | Dacl |

**Supplementary Table 3B.** League table for clinical response in induction therapy.

| Vedoli |  |  |  |  |  |  |  |  |  |  |  |  |  |  |  |  |
| --- | --- | --- | --- | --- | --- | --- | --- | --- | --- | --- | --- | --- | --- | --- | --- | --- |
| 1.74 (0.43,7.06) | Inflix+AZA |  |  |  |  |  |  |  |  |  |  |  |  |  |  |  |
| 2.46 (0.74,8.18) | 1.42 (0.69,2.91) | Inflix |  |  |  |  |  |  |  |  |  |  |  |  |  |  |
| **3.37 (1.01,11.19)** | 1.94 (0.81,4.62) | 1.37 (0.84,2.23) | Usteki |  |  |  |  |  |  |  |  |  |  |  |  |  |
| **3.64 (1.09,12.16)** | 2.09 (0.87,5.02) | 1.48 (0.90,2.43) | 1.08 (0.65,1.78) | Golimu |  |  |  |  |  |  |  |  |  |  |  |  |
| **4.40 (1.21,16.04)** | 2.53 (0.94,6.83) | 1.79 (0.90,3.54) | 1.31 (0.66,2.58) | 1.21 (0.61,2.42) | PF-00547659 |  |  |  |  |  |  |  |  |  |  |  |
| **4.67 (1.16,18.90)** | 2.69 (0.87,8.28) | 1.90 (0.80,4.51) | 1.39 (0.58,3.29) | 1.29 (0.54,3.08) | 1.06 (0.40,2.85) | BMS-936557 |  |  |  |  |  |  |  |  |  |  |
| **5.23 (1.44,19.03)** | **3.01 (1.12,8.10)** | **2.13 (1.08,4.20)** | 1.55 (0.79,3.06) | 1.44 (0.72,2.87) | 1.19 (0.52,2.73) | 1.12 (0.42,3.00) | Elde |  |  |  |  |  |  |  |  |  |
| **5.35 (1.35,21.24)** | **3.08 (1.53,6.18)** | **2.17 (1.11,4.27)** | 1.59 (0.69,3.65) | 1.47 (0.64,3.40) | 1.22 (0.46,3.18) | 1.14 (0.38,3.43) | 1.02 (0.39,2.67) | AZA |  |  |  |  |  |  |  |  |
| **5.66 (1.72,18.63)** | **3.26 (1.38,7.66)** | **2.30 (1.45,3.65)** | **1.68 (1.06,2.66)** | 1.56 (0.97,2.49) | 1.29 (0.66,2.50) | 1.21 (0.52,2.84) | 1.08 (0.56,2.10) | 1.06 (0.47,2.40) | Etrol |  |  |  |  |  |  |  |
| **5.77 (1.77,18.74)** | **3.32 (1.43,7.67)** | **2.34 (1.53,3.60)** | **1.71 (1.12,2.62)** | **1.59 (1.02,2.46)** | 1.31 (0.69,2.49) | 1.23 (0.54,2.84) | 1.10 (0.58,2.09) | 1.08 (0.48,2.40) | 1.02 (0.74,1.40) | Adal |  |  |  |  |  |  |
| **6.62 (1.62,27.06)** | **3.81 (1.22,11.85)** | **2.69 (1.12,6.47)** | 1.97 (0.82,4.74) | 1.82 (0.88,3.75) | 1.50 (0.55,4.10) | 1.42 (0.46,4.40) | 1.27 (0.47,3.44) | 1.24 (0.41,3.75) | 1.17 (0.49,2.77) | 1.15 (0.49,2.68) | Guselku |  |  |  |  |  |
| **6.90 (1.74,27.41)** | **3.97 (1.32,11.96)** | **2.80 (1.21,6.47)** | 2.05 (0.89,4.72) | 1.90 (0.82,4.41) | 1.57 (0.60,4.10) | 1.48 (0.49,4.43) | 1.32 (0.50,3.45) | 1.29 (0.44,3.78) | 1.22 (0.54,2.77) | 1.20 (0.54,2.67) | 1.04 (0.34,3.17) | Visil |  |  |  |  |
| **11.23 (2.78,45.35)** | **6.46 (2.10,19.87)** | **4.56 (1.92,10.81)** | **3.33 (1.41,7.88)** | **3.09 (1.29,7.38)** | 2.55 (0.95,6.84) | 2.40 (0.78,7.36) | 2.15 (0.80,5.74) | 2.10 (0.70,6.28) | 1.98 (0.85,4.63) | 1.95 (0.85,4.47) | 1.70 (0.55,5.26) | 1.63 (0.54,4.88) | Basil |  |  |  |
| **11.26 (2.88,44.03)** | **6.48 (2.19,19.13)** | **4.58 (2.04,10.28)** | **3.34 (1.49,7.49)** | **3.10 (1.37,7.01)** | 2.56 (1.00,6.54) | 2.41 (0.82,7.09) | 2.15 (0.84,5.49) | 2.10 (0.73,6.04) | 1.99 (0.90,4.39) | 1.95 (0.90,4.24) | 1.70 (0.57,5.07) | 1.63 (0.57,4.69) | 1.00 (0.34,2.94) | Cobit |  |  |
| **18.84 (4.89,72.58)** | **10.84 (3.74,31.41)** | **7.66 (3.50,16.77)** | **5.59 (2.56,12.22)** | **5.18 (2.35,11.45)** | **4.28 (1.71,10.71)** | **4.03 (1.40,11.64)** | **3.60 (1.44,8.99)** | **3.52 (1.25,9.91)** | **3.33 (1.54,7.17)** | **3.27 (1.55,6.91)** | 2.84 (0.97,8.32) | 2.73 (0.97,7.70) | 1.68 (0.58,4.84) | 1.67 (0.61,4.61) | Dacl |  |
| **9.60 (3.04,30.35)** | **5.52 (2.48,12.28)** | **3.90 (2.76,5.52)** | **2.85 (2.02,4.01)** | **2.64 (1.83,3.80)** | **2.18 (1.21,3.93)** | 2.05 (0.93,4.54) | **1.83 (1.02,3.30)** | 1.79 (0.84,3.83) | **1.70 (1.25,2.30)** | **1.67 (1.29,2.15)** | 1.45 (0.64,3.26) | 1.39 (0.65,2.98) | 0.86 (0.39,1.89) | 0.85 (0.41,1.77) | 0.51 (0.25,1.03) | Placebo |

**Supplementary Table 3C.** League table for endoscopic improve in induction therapy.

| Usteki |  |  |  |  |
| --- | --- | --- | --- | --- |
| 1.34 (0.82,2.19) | Etrol |  |  |  |
| 1.43 (0.84,2.44) | 1.07 (0.77,1.48) | Adal |  |  |
| **3.04 (1.30,7.14)** | 2.28 (0.98,5.28) | 2.13 (0.89,5.05) | Cobit |  |
| **2.31 (1.60,3.31)** | **1.72 (1.23,2.41)** | **1.61 (1.09,2.38)** | 0.76 (0.35,1.64) | Placebo |

**Supplementary Table 3D.** League table for mucosal healing in induction therapy.

| Inflix + AZA |  |  |  |  |  |  |  |  |  |  |  |  |
| --- | --- | --- | --- | --- | --- | --- | --- | --- | --- | --- | --- | --- |
| 1.11 (0.52,2.37) | Inflix |  |  |  |  |  |  |  |  |  |  |  |
| 1.69 (0.60,4.70) | 1.52 (0.76,3.03) | Adal |  |  |  |  |  |  |  |  |  |  |
| 1.74 (0.60,5.06) | 1.57 (0.75,3.33) | 1.03 (0.62,1.72) | Etrol |  |  |  |  |  |  |  |  |  |
| 1.91 (0.89,4.14) | 1.73 (0.80,3.75) | 1.14 (0.40,3.20) | 1.10 (0.37,3.22) | AZA |  |  |  |  |  |  |  |  |
| 2.60 (0.73,9.22) | 2.35 (0.85,6.46) | 1.54 (0.56,4.21) | 1.49 (0.52,4.24) | 1.36 (0.38,4.85) | BMS-936557 |  |  |  |  |  |  |  |
| 2.96 (0.80,10.89) | 2.67 (0.93,7.70) | 1.75 (0.61,5.02) | 1.70 (0.57,5.04) | 1.54 (0.42,5.73) | 1.14 (0.31,4.15) | Visil |  |  |  |  |  |  |
| 2.97 (0.93,9.46) | **2.68 (1.12,6.43)** | 1.76 (0.74,4.18) | 1.70 (0.69,4.23) | 1.55 (0.48,4.98) | 1.14 (0.36,3.60) | 1.00 (0.31,3.29) | Elde |  |  |  |  |  |
| 3.35 (0.98,11.44) | **3.03 (1.16,7.94)** | 1.99 (0.76,5.17) | 1.92 (0.71,5.21) | 1.75 (0.51,6.02) | 1.29 (0.38,4.36) | 1.13 (0.32,3.98) | 1.13 (0.37,3.41) | Basil |  |  |  |  |
| **3.89 (1.42,10.65)** | **3.51 (1.82,6.80)** | **2.31 (1.23,4.33)** | **2.23 (1.12,4.45)** | 2.03 (0.73,5.62) | 1.50 (0.56,4.03) | 1.32 (0.47,3.71) | 1.31 (0.56,3.06) | 1.16 (0.45,2.97) | Golimu |  |  |  |
| **5.30 (1.66,16.89)** | **4.79 (2.00,11.49)** | **3.14 (1.32,7.47)** | **3.04 (1.22,7.56)** | 2.77 (0.86,8.90) | 2.04 (0.65,6.43) | 1.79 (0.55,5.88) | 1.79 (0.64,4.99) | 1.58 (0.52,4.78) | 1.36 (0.58,3.19) | PF-00547659 |  |  |
| **9.55 (2.00,45.61)** | **8.63 (2.20,33.83)** | **5.67 (1.47,21.89)** | **5.48 (1.38,21.79)** | **4.99 (1.04,23.97)** | 3.68 (0.78,17.37) | 3.23 (0.66,15.73) | 3.22 (0.74,13.95) | 2.85 (0.62,13.05) | 2.46 (0.74,8.12) | 1.80 (0.42,7.82) | Guselku |  |
| **3.44 (1.39,8.49)** | **3.11 (1.91,5.06)** | **2.04 (1.28,3.26)** | **1.97 (1.14,3.42)** | 1.80 (0.72,4.48) | 1.32 (0.54,3.22) | 1.16 (0.45,2.98) | 1.16 (0.56,2.40) | 1.03 (0.45,2.36) | 0.88 (0.57,1.37) | 0.65 (0.31,1.34) | 0.36 (0.10,1.29) | Placebo |

**Supplementary Figure 1.** Scatter plots of relative treatment rankings per SUCRA estimates for selected outcome combinations in induction therapy.

**
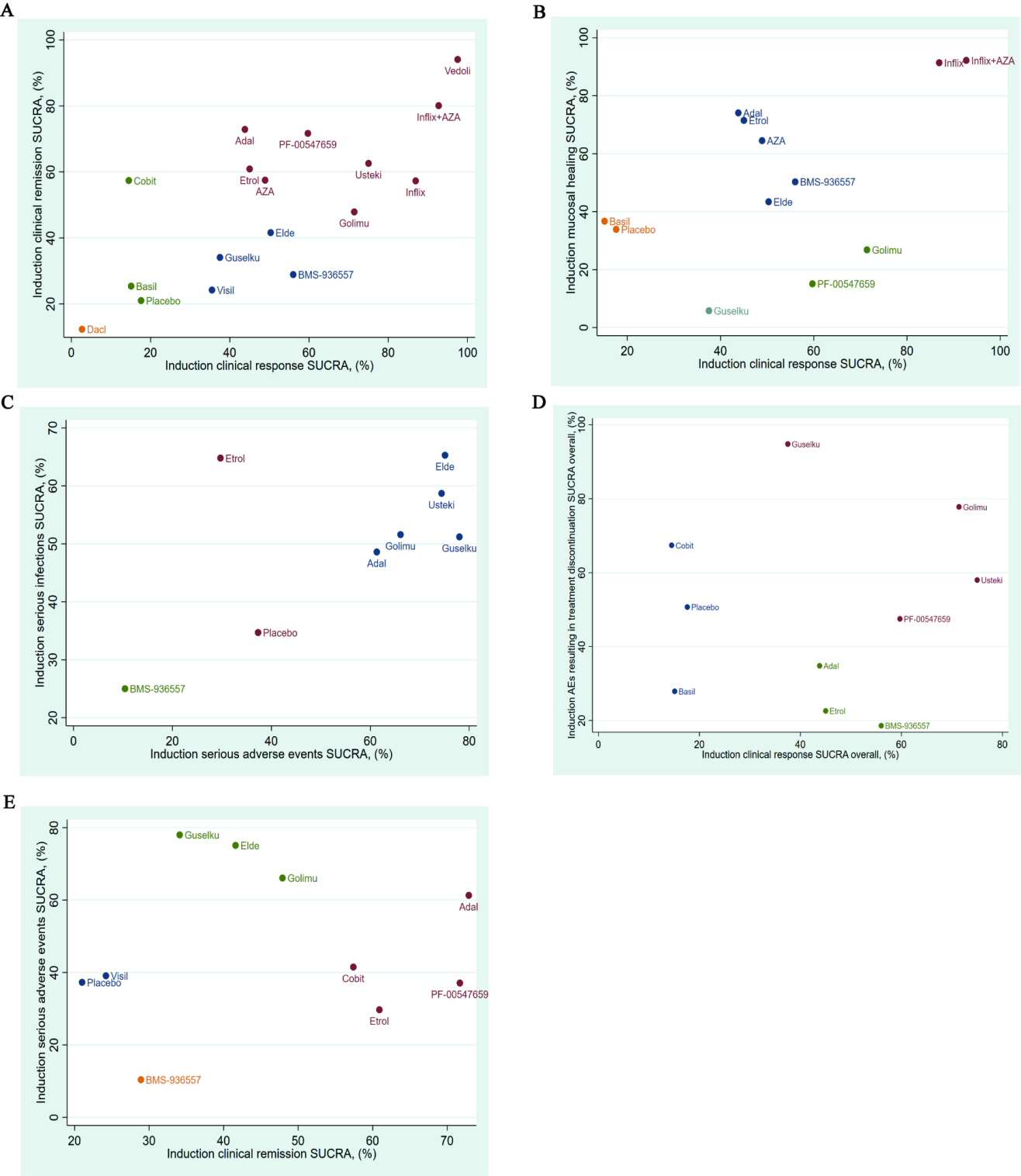
**

A: SUCRA estimates for clinical response vs clinical remission in induction therapy. B: SUCRA estimates for endoscopic improve vs clinical remission in induction therapy. C: SUCRA estimates for serious infections vs serious AEs in induction therapy. D: SUCRA estimates for discontinuation due to AEss vs clinical remission in induction therapy. E: SUCRA estimates for serious AEs vs clinical remission in induction therapy.

**Supplementary Table 4A.** League table for clinical remission in maintenance therapy.

| Vedoli |  |  |  |  |  |  |  |  |
| --- | --- | --- | --- | --- | --- | --- | --- | --- |
| 5.26 (0.88,31.46) | Inflix |  |  |  |  |  |  |  |
| 2.56 (0.65,10.07) | 0.49 (0.08,2.91) | Adal |  |  |  |  |  |  |
| 3.53 (0.66,19.05) | 0.67 (0.09,5.16) | 1.38 (0.26,7.45) | Golimu |  |  |  |  |  |
| 2.25 (0.60,8.39) | 0.43 (0.07,2.45) | 0.88 (0.24,3.28) | 0.64 (0.22,1.82) | Etrol |  |  |  |  |
| **3.17 (1.01,9.96)** | 0.60 (0.12,3.06) | 1.24 (0.39,3.90) | 0.90 (0.20,4.06) | 1.41 (0.48,4.16) | Usteki |  |  |  |
| 1.38 (0.44,4.32) | 0.26 (0.05,1.32) | 0.54 (0.17,1.69) | 0.39 (0.09,1.76) | 0.62 (0.21,1.80) | 0.44 (0.19,1.02) | Guselku |  |  |
| **4.36 (1.65,11.49)** | 0.83 (0.18,3.72) | 1.70 (0.65,4.49) | 1.23 (0.31,4.89) | 1.94 (0.79,4.73) | 1.37 (0.75,2.53) | 3**.15 (1.73,5.73)** | Mesalazine |  |
| 1.61 (0.48,5.38) | 0.31 (0.08,1.15) | 0.63 (0.19,2.10) | 0.46 (0.10,2.15) | 0.72 (0.23,2.25) | 0.51 (0.20,1.30) | 1.16 (0.46,2.95) | **0.37 (0.18,0.76)** | Placebo |

**Supplementary Table 4B.** League table for clinical response in maintenance therapy.

| Inflix |  |  |  |  |  |  |  |  |
| --- | --- | --- | --- | --- | --- | --- | --- | --- |
| 1.30 (0.10,17.05) | Usteki |  |  |  |  |  |  |  |
| 1.77 (0.13,23.29) | 1.36 (0.06,31.09) | Golimu |  |  |  |  |  |  |
| 1.90 (0.14,25.11) | 1.47 (0.06,33.52) | 1.08 (0.05,24.71) | Adal |  |  |  |  |  |
| 2.05 (0.06,76.00) | 1.58 (0.03,88.28) | 1.16 (0.02,65.05) | 1.08 (0.02,60.39) | Vedoli |  |  |  |  |
| 3.22 (0.10,101.03) | 2.48 (0.05,119.46) | 1.82 (0.19,17.93) | 1.69 (0.03,81.69) | 1.57 (0.02,161.31) | Guselku |  |  |  |
| 3.58 (0.27,48.14) | 2.76 (0.12,64.05) | 2.03 (0.09,47.23) | 1.88 (0.08,43.86) | 1.75 (0.03,99.41) | 1.11 (0.02,54.51) | Etrol |  |  |
| 11.42 (0.31,424.29) | 8.80 (0.16,492.81) | 6.47 (0.12,363.03) | 6.00 (0.48,75.50) | 5.58 (0.05,649.77) | 3.55 (0.03,364.49) | 3.19 (0.06,181.16) | Mesalazine |  |
| 3.69 (0.98,13.82) | 2.84 (0.31,25.88) | 2.09 (0.23,19.11) | 1.94 (0.21,17.76) | 1.80 (0.06,52.02) | 1.15 (0.05,27.64) | 1.03 (0.11,9.63) | 0.32 (0.01,9.34) | Placebo |

**Supplementary Table 4C.** League table for endoscopic improve in maintenance therapy.

| Vedoli |  |  |  |
| --- | --- | --- | --- |
| 1.88 (0.53,6.67) | Etrol |  |  |
| 3.02 (0.88,10.41) | 1.60 (0.57,4.49) | Usteki |  |
| **4.05 (1.46,11.19)** | **2.15 (1.01,4.54)** | 1.34 (0.66,2.72) | Placebo |

**Supplementary Table 4D.** League table for mucosal healing in maintenance therapy.

| Inflix |  |  |  |  |  |
| --- | --- | --- | --- | --- | --- |
| 1.16 (0.62,2.19) | Etrol |  |  |  |  |
| 1.22 (0.63,2.33) | 1.05 (0.51,2.15) | Adal |  |  |  |
| 1.35 (0.70,2.59) | 1.16 (0.56,2.41) | 1.11 (0.53,2.31) | Golimu |  |  |
| 3.31 (0.87,12.51) | 2.84 (0.72,11.21) | 2.72 (0.69,10.74) | 2.46 (0.77,7.83) | Guselku |  |
| **2.72 (1.86,3.98)** | **2.34 (1.41,3.88)** | **2.23 (1.34,3.73)** | **2.02 (1.19,3.43)** | 0.82 (0.23,2.94) | Placebo |

**Supplementary Figure 2.** Scatter plots of relative treatment rankings per SUCRA estimates for selected outcome combinations in maintenance therapy.


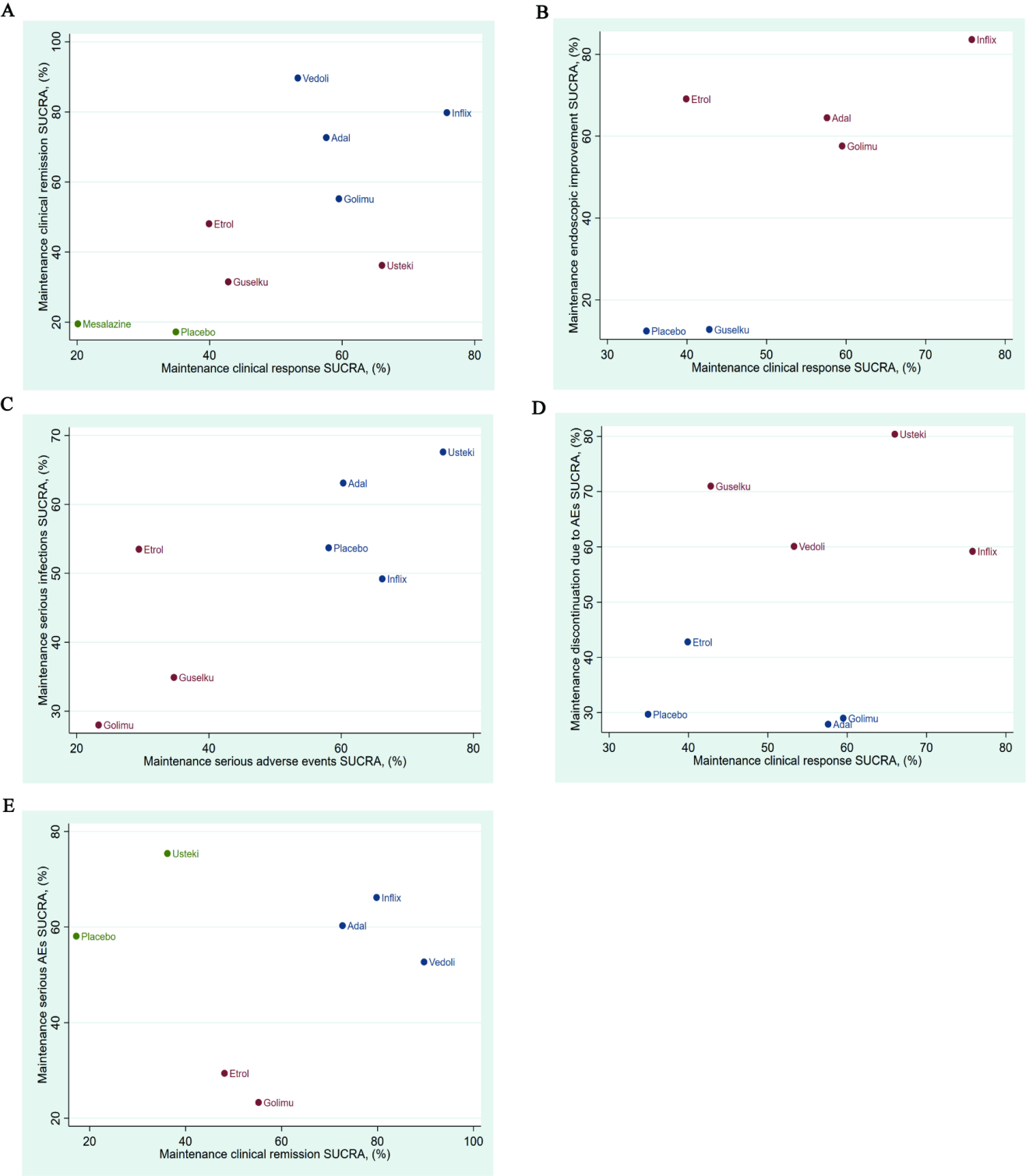


A: SUCRA estimates for clinical response vs clinical remission in maintenance therapy. B: SUCRA estimates for endoscopic improve vs clinical remission in maintenance therapy. C: SUCRA estimates for serious infections vs serious AEs in maintenance therapy. D: SUCRA estimates for discontinuation due to AEss vs clinical remission in maintenance therapy. E: SUCRA estimates for serious AEs vs clinical remission in maintenance therapy.

**Supplementary Table 5A.** League table for adverse events in induction therapy.

| Cobit |  |  |  |  |  |  |  |  |  |  |  |
| --- | --- | --- | --- | --- | --- | --- | --- | --- | --- | --- | --- |
| 0.89 (0.34,2.36) | Guselku |  |  |  |  |  |  |  |  |  |  |
| 0.67 (0.32,1.37) | 0.75 (0.35,1.58) | Usteki |  |  |  |  |  |  |  |  |  |
| 0.65 (0.31,1.35) | 0.73 (0.34,1.55) | 0.98 (0.66,1.44) | Etrol |  |  |  |  |  |  |  |  |
| 0.62 (0.26,1.48) | 0.69 (0.28,1.70) | 0.92 (0.49,1.73) | 0.95 (0.50,1.78) | Elde |  |  |  |  |  |  |  |
| 0.59 (0.29,1.20) | 0.66 (0.34,1.27) | 0.88 (0.61,1.27) | 0.90 (0.62,1.31) | 0.95 (0.51,1.77) | Golimu |  |  |  |  |  |  |
| 0.58 (0.28,1.18) | 0.65 (0.31,1.37) | 0.87 (0.60,1.25) | 0.89 (0.67,1.19) | 0.94 (0.51,1.75) | 0.99 (0.70,1.40) | Adal |  |  |  |  |  |
| 0.53 (0.19,1.46) | 0.59 (0.21,1.67) | 0.79 (0.35,1.78) | 0.81 (0.36,1.83) | 0.85 (0.33,2.22) | 0.90 (0.40,2.00) | 0.91 (0.41,2.03) | Basil |  |  |  |  |
| 0.52 (0.22,1.22) | 0.59 (0.24,1.40) | 0.79 (0.44,1.41) | 0.80 (0.45,1.45) | 0.85 (0.39,1.83) | 0.89 (0.50,1.58) | 0.90 (0.51,1.60) | 1.00 (0.39,2.51) | PF-00547659 |  |  |  |
| 0.46 (0.17,1.29) | 0.52 (0.18,1.48) | 0.70 (0.31,1.58) | 0.71 (0.31,1.63) | 0.75 (0.29,1.97) | 0.79 (0.35,1.78) | 0.80 (0.36,1.81) | 0.88 (0.30,2.63) | 0.89 (0.35,2.25) | BMS-936557 |  |  |
| 0.08 (0.02,0.34) | 0.08 (0.02,0.39) | 0.11 (0.03,0.45) | **0.12 (0.03,0.46)** | **0.12 (0.03,0.53)** | **0.13 (0.03,0.51)** | **0.13 (0.03,0.51)** | **0.14 (0.03,0.68)** | **0.14 (0.03,0.61)** | 0.16 (0.03,0.77) | Visil |  |
| 0.61 (0.31,1.20) | 0.69 (0.34,1.39) | 0.92 (0.70,1.21) | 0.94 (0.71,1.25) | 1.00 (0.57,1.76) | 1.05 (0.82,1.34) | 1.06 (0.83,1.36) | 1.17 (0.54,2.52) | 1.17 (0.70,1.97) | **1.32 (0.61,2.87)** | **8.18 (2.12,31.63)** | Placebo |

**Supplementary Table 5B.** League table for recurrence of ulcerative colitis in induction therapy.

| Guselku |  |  |  |  |  |  |  |  |
| --- | --- | --- | --- | --- | --- | --- | --- | --- |
| 0.15 (0.01,2.52) | Usteki |  |  |  |  |  |  |  |
| 0.13 (0.01,2.06) | 0.90 (0.19,4.16) | Adal |  |  |  |  |  |  |
| **0.02 (0.00,0.47)** | 0.15 (0.02,1.16) | 0.17 (0.03,1.10) | Dacl |  |  |  |  |  |
| 0.12 (0.01,1.82) | 0.83 (0.19,3.63) | 0.92 (0.33,2.59) | 5.49 (0.87,34.48) | Etrol |  |  |  |  |
| 0.11 (0.01,2.47) | 0.77 (0.09,6.27) | 0.86 (0.12,6.01) | 5.11 (0.48,54.28) | 0.93 (0.14,6.25) | PF-00547659 |  |  |  |
| **0.10 (0.01,0.99)** | 0.66 (0.13,3.34) | 0.73 (0.17,3.15) | 4.35 (0.61,30.85) | 0.79 (0.20,3.15) | 0.85 (0.11,6.43) | Golimu |  |  |
| **0.06 (0.00,1.20)** | 0.43 (0.06,2.84) | 0.48 (0.09,2.68) | 2.84 (0.32,25.22) | 0.52 (0.10,2.77) | 0.56 (0.06,5.22) | 0.65 (0.11,3.97) | Cobit |  |
| **0.07 (0.01,0.87)** | 0.45 (0.13,1.53) | 0.51 (0.20,1.29) | 3.01 (0.59,15.43) | 0.55 (0.24,1.27) | 0.59 (0.11,3.25) | 0.69 (0.24,2.03) | 1.06 (0.25,4.52) | Placebo |

**Supplementary Table 5C.** League table for infections in induction therapy.

| Etrol |  |  |  |  |  |  |  |  |
| --- | --- | --- | --- | --- | --- | --- | --- | --- |
| 0.98 (0.25,3.87) | Guselku |  |  |  |  |  |  |  |
| 0.91 (0.55,1.51) | 0.93 (0.23,3.69) | Usteki |  |  |  |  |  |  |
| 0.88 (0.61,1.27) | 0.89 (0.23,3.46) | 0.96 (0.61,1.52) | Adal |  |  |  |  |  |
| 0.78 (0.37,1.66) | 0.80 (0.18,3.53) | 0.86 (0.40,1.84) | 0.89 (0.44,1.83) | PF-00547659 |  |  |  |  |
| 0.75 (0.35,1.58) | 0.76 (0.17,3.36) | 0.82 (0.38,1.76) | 0.85 (0.41,1.75) | 0.95 (0.37,2.44) | Elde |  |  |  |
| 0.54 (0.19,1.57) | 0.55 (0.23,1.32) | 0.60 (0.20,1.74) | 0.62 (0.22,1.75) | 0.69 (0.21,2.32) | 0.73 (0.22,2.43) | Golimu |  |  |
| 0.38 (0.09,1.63) | 0.39 (0.06,2.70) | 0.42 (0.10,1.80) | 0.44 (0.10,1.83) | 0.49 (0.10,2.32) | 0.51 (0.11,2.44) | 0.70 (0.12,3.97) | BMS-936557 |  |
| 0.95 (0.67,1.34) | 0.97 (0.26,3.65) | 1.04 (0.72,1.51) | 1.08 (0.83,1.41) | 1.21 (0.62,2.35) | 1.27 (0.65,2.48) | 1.74 (0.64,4.76) | 2.48 (0.61,10.14) | Placebo |

**Supplementary Table 5D.** League table for adverse events resulting in treatment discontinuation in induction therapy.

| Guselku |  |  |  |  |  |  |  |  |  |
| --- | --- | --- | --- | --- | --- | --- | --- | --- | --- |
| 0.41 (0.13,1.24) | Golimu |  |  |  |  |  |  |  |  |
| 0.22 (0.02,2.82) | 0.55 (0.06,5.36) | Cobit |  |  |  |  |  |  |  |
| 0.16 (0.02,1.70) | 0.40 (0.05,3.15) | 0.72 (0.13,4.08) | Usteki |  |  |  |  |  |  |
| 0.12 (0.01,1.59) | 0.28 (0.03,3.05) | 0.51 (0.06,4.16) | 0.71 (0.11,4.56) | PF-00547659 |  |  |  |  |  |
| 0.07 (0.00,1.40) | 0.16 (0.01,2.79) | 0.29 (0.02,4.00) | 1.01 (0.05,19.37) | 0.57 (0.04,8.48) | Adal |  |  |  |  |
| 0.05 (0.00,1.04) | 0.12 (0.01,2.06) | 0.22 (0.02,2.95) | 0.31 (0.03,3.39) | 0.43 (0.03,6.26) | 0.76 (0.03,16.75) | Basil |  |  |  |
| 0.04 (0.00,0.88) | 0.11 (0.01,1.75) | 0.20 (0.02,2.50) | 0.27 (0.03,2.87) | 0.38 (0.03,5.32) | 0.67 (0.19,2.39) | 0.88 (0.04,18.48) | Etrol |  |  |
| 0.03 (0.00,1.09) | 0.06 (0.00,2.25) | 0.12 (0.00,3.39) | 0.16 (0.01,4.08) | 0.23 (0.01,7.06) | 0.40 (0.01,17.35) | 0.53 (0.01,22.52) | 0.60 (0.01,24.92) | BMS-936557 |  |
| 0.13 (0.02,1.11) | 0.33 (0.05,1.99) | 0.60 (0.15,2.41) | 0.83 (0.30,2.29) | 1.16 (0.25,5.50) | 0.40 (0.03,5.10) | 2.69 (0.31,23.65) | 3.05 (0.36,25.66) | 5.09 (0.24,108.59) | Placebo |

**Supplementary Table 5E.** League table for serious adverse events in induction therapy.

| Guselku |  |  |  |  |  |  |  |  |  |  |
| --- | --- | --- | --- | --- | --- | --- | --- | --- | --- | --- |
| 0.63 (0.04,10.85) | Elde |  |  |  |  |  |  |  |  |  |
| 0.58 (0.04,8.93) | 0.93 (0.23,3.79) | Usteki |  |  |  |  |  |  |  |  |
| 0.49 (0.04,5.82) | 0.77 (0.19,3.13) | 0.83 (0.27,2.58) | Golimu |  |  |  |  |  |  |  |
| 0.43 (0.03,6.57) | 0.69 (0.18,2.58) | 0.74 (0.26,2.09) | 0.89 (0.29,2.70) | Adal |  |  |  |  |  |  |
| 0.28 (0.01,6.18) | 0.44 (0.06,3.39) | 0.48 (0.07,3.07) | 0.57 (0.09,3.66) | 0.64 (0.11,3.88) | Cobit |  |  |  |  |  |
| 0.28 (0.02,4.87) | 0.45 (0.09,2.27) | 0.49 (0.12,1.95) | 0.59 (0.15,2.33) | 0.66 (0.18,2.41) | 1.02 (0.14,7.70) | Visil |  |  |  |  |
| 0.27 (0.01,4.78) | 0.42 (0.08,2.31) | 0.46 (0.10,2.01) | 0.55 (0.13,2.40) | 0.62 (0.15,2.50) | 0.96 (0.12,7.70) | 0.94 (0.17,5.02) | PF-00547659 |  |  |  |
| 0.24 (0.02,3.69) | 0.39 (0.10,1.52) | 0.42 (0.14,1.24) | 0.50 (0.17,1.52) | 0.56 (0.24,1.30) | 0.87 (0.14,5.44) | 0.86 (0.22,3.29) | 0.91 (0.22,3.87) | Etrol |  |  |
| 0.07 (0.00,2.26) | 0.11 (0.01,1.45) | 0.12 (0.01,1.37) | 0.14 (0.01,1.63) | 0.16 (0.02,1.75) | 0.25 (0.01,4.31) | 0.25 (0.02,3.18) | 0.26 (0.02,3.57) | 0.29 (0.03,3.19) | BMS-936557 |  |
| 0.29 (0.02,3.96) | 0.47 (0.15,1.47) | 0.50 (0.22,1.12) | 0.60 (0.27,1.33) | 0.68 (0.35,1.29) | 1.05 (0.20,5.61) | 1.03 (0.33,3.18) | 1.10 (0.32,3.81) | 1.20 (0.58,2.49) | 4.16 (0.42,41.00) | Placebo |

**Supplementary Table 5F.** League table for serious infections in induction therapy.

| Elde |  |  |  |  |  |  |  |
| --- | --- | --- | --- | --- | --- | --- | --- |
| 0.67 (0.00,180.30) | Etrol |  |  |  |  |  |  |
| 0.55 (0.00,303.35) | 0.82 (0.01,99.58) | Usteki |  |  |  |  |  |
| 0.33 (0.00,1340.44) | 0.50 (0.00,676.19) | 0.60 (0.00,1459.15) | Guselku |  |  |  |  |
| 0.33 (0.00,178.95) | 0.49 (0.00,58.56) | 0.60 (0.00,162.75) | 0.99 (0.00,219.25) | Golimu |  |  |  |
| 0.28 (0.00,67.81) | 0.42 (0.02,10.37) | 0.51 (0.00,54.67) | 0.85 (0.00,1063.29) | 0.86 (0.01,90.46) | Adal |  |  |
| 0.05 (0.00,37.51) | 0.08 (0.00,13.26) | 0.10 (0.00,35.17) | 0.16 (0.00,471.91) | 0.16 (0.00,58.36) | 0.19 (0.00,27.80) | BMS-936557 |  |
| 0.16 (0.00,21.87) | 0.24 (0.02,3.56) | 0.29 (0.01,15.70) | 0.49 (0.00,395.85) | 0.49 (0.01,25.91) | 0.57 (0.05,6.62) | 3.06 (0.04,238.97) | Placebo |

**Supplementary Table 6A.** League table for adverse events in maintenance therapy.

| Vedoli |  |  |  |  |  |  |  |
| --- | --- | --- | --- | --- | --- | --- | --- |
| 0.43 (0.09,2.08) | Etrol |  |  |  |  |  |  |
| 0.77 (0.18,3.25) | 1.80 (0.44,7.40) | Usteki |  |  |  |  |  |
| 0.84 (0.20,3.52) | 1.96 (0.48,8.00) | 1.09 (0.31,3.79) | Adal |  |  |  |  |
| 0.30 (0.04,2.18) | 0.69 (0.21,2.34) | 0.39 (0.06,2.49) | 0.35 (0.06,2.27) | Golimu |  |  |  |
| 0.61 (0.20,1.88) | 1.41 (0.47,4.25) | 0.79 (0.32,1.91) | 0.72 (0.30,1.72) | 2.04 (0.39,10.50) | Inflix |  |  |
| 0.45 (0.12,1.71) | 1.05 (0.29,3.90) | 0.59 (0.19,1.82) | 0.54 (0.18,1.65) | 1.52 (0.26,9.05) | 0.75 (0.37,1.51) | Guselku |  |
| 0.46 (0.11,1.82) | 1.06 (0.27,4.14) | 0.59 (0.18,1.95) | 0.54 (0.17,1.77) | 1.53 (0.25,9.47) | 0.75 (0.34,1.67) | 1.01 (0.35,2.91) | Placebo |

**Supplementary Table 6B.** League table for recurrence of ulcerative colitis in maintenance therapy.

| Guselku |  |  |  |  |  |  |  |
| --- | --- | --- | --- | --- | --- | --- | --- |
| 0.71 (0.19,2.71) | Usteki |  |  |  |  |  |  |
| 0.38 (0.10,1.41) | **0.53 (0.28,0.99)** | Etrol |  |  |  |  |  |
| 0.32 (0.08,1.17) | **0.44 (0.24,0.81)** | 0.84 (0.47,1.49) | Inflix |  |  |  |  |
| 0.28 (0.07,1.11) | **0.39 (0.18,0.83)** | 0.74 (0.35,1.53) | 0.88 (0.43,1.80) | Vedoli |  |  |  |
| 0.27 (0.06,1.14) | **0.37 (0.16,0.89)** | 0.71 (0.30,1.66) | 0.85 (0.37,1.95) | 0.96 (0.37,2.47) | Adal |  |  |
| **0.19 (0.06,0.59)** | **0.26 (0.13,0.52)** | **0.50 (0.26,0.96)** | 0.59 (0.31,1.12) | 0.67 (0.31,1.47) | 0.70 (0.29,1.71) | Golimu |  |
| **0.16 (0.05,0.57)** | **0.23 (0.14,0.36)** | **0.43 (0.28,0.66)** | **0.52 (0.35,0.76)** | 0.59 (0.32,1.06) | 0.61 (0.29,1.28) | 0.87 (0.53,1.44) | Placebo |

**Supplementary Table 6C.** League table for infections in maintenance therapy.

| Usteki |  |  |  |  |  |  |  |
| --- | --- | --- | --- | --- | --- | --- | --- |
| 0.63 (0.39,1.04) | Guselku |  |  |  |  |  |  |
| 0.18 (0.03,1.16) | 0.29 (0.05,1.83) | Vedoli |  |  |  |  |  |
| 0.50 (0.29,0.87) | 0.79 (0.47,1.35) | 2.75 (0.43,17.80) | Etrol |  |  |  |  |
| 0.60 (0.35,1.03) | 0.95 (0.57,1.59) | 3.31 (0.51,21.24) | 1.20 (0.68,2.12) | Adal |  |  |  |
| 0.82 (0.57,1.18) | 1.30 (0.93,1.81) | 4.50 (0.73,27.74) | 1.63 (1.08,2.48) | 1.36 (0.92,2.01) | Inflix |  |  |
| 0.67 (0.42,1.07) | 1.06 (0.68,1.65) | 3.67 (0.58,23.16) | 1.33 (0.80,2.22) | 1.11 (0.68,1.81) | 0.82 (0.61,1.10) | Placebo |  |
| 0.78 (0.31,1.96) | 1.23 (0.50,3.05) | 4.27 (0.57,31.75) | 1.55 (0.74,3.24) | 1.29 (0.51,3.28) | 0.95 (0.41,2.21) | 1.16 (0.47,2.86) | Golimu |

**Supplementary Table 6D.** League table for adverse events resulting in treatment discontinuation in maintenance therapy.

| Usteki |  |  |  |  |  |  |  |
| --- | --- | --- | --- | --- | --- | --- | --- |
| 0.51 (0.08,3.06) | Guselku |  |  |  |  |  |  |
| 0.57 (0.06,5.84) | 1.12 (0.11,11.21) | Vedoli |  |  |  |  |  |
| 0.24 (0.03,2.01) | 0.46 (0.06,3.86) | 0.41 (0.03,5.52) | Etrol |  |  |  |  |
| 0.34 (0.05,2.15) | 0.67 (0.11,4.09) | 0.60 (0.06,6.24) | 1.45 (0.17,12.56) | Adal |  |  |  |
| **0.27 (0.07,0.96)** | 0.52 (0.15,1.81) | 0.47 (0.07,3.25) | 1.13 (0.20,6.26) | 0.78 (0.21,2.86) | Inflix |  |  |
| 1.00 (0.03,31.31) | 1.96 (0.06,60.49) | 1.76 (0.04,73.63) | 4.24 (0.29,62.69) | 2.92 (0.09,91.92) | 3.76 (0.15,91.69) | Golimu |  |
| 0.24 (0.04,1.38) | 0.47 (0.08,2.66) | 0.42 (0.04,4.12) | 1.01 (0.12,8.26) | 0.70 (0.12,4.14) | 0.90 (0.27,3.01) | 0.24 (0.01,7.26) | Placebo |

**Supplementary Table 6E.** League table for serious adverse events in maintenance therapy.

| Golimu |  |  |  |  |  |  |  |
| --- | --- | --- | --- | --- | --- | --- | --- |
| 0.99 (0.24,4.10) | Etrol |  |  |  |  |  |  |
| 1.65 (0.32,8.61) | 1.68 (0.73,3.86) | Guselku |  |  |  |  |  |
| 1.42 (0.23,8.87) | 1.44 (0.46,4.55) | 0.86 (0.31,2.42) | Vedoli |  |  |  |  |
| 1.08 (0.19,6.07) | 1.10 (0.41,2.90) | 0.65 (0.28,1.51) | 0.76 (0.24,2.40) | Adal |  |  |  |
| 1.52 (0.31,7.41) | 1.54 (0.78,3.07) | 0.92 (0.57,1.48) | 1.07 (0.43,2.68) | 1.41 (0.71,2.82) | Inflix |  |  |
| 1.87 (0.34,10.28) | 1.90 (0.75,4.83) | 1.13 (0.51,2.50) | 1.32 (0.43,4.01) | 1.73 (0.68,4.42) | 1.23 (0.65,2.31) | Usteki |  |
| 1.56 (0.29,8.25) | 1.58 (0.67,3.75) | 0.94 (0.46,1.91) | 1.09 (0.38,3.16) | 1.44 (0.60,3.44) | 1.02 (0.60,1.73) | 0.83 (0.37,1.89) | Placebo |

**Supplementary Table 6F.** League table for serious infections in induction therapy.

| Usteki |  |  |  |  |  |  |
| --- | --- | --- | --- | --- | --- | --- |
| 0.76 (0.13,4.55) | Adal |  |  |  |  |  |
| 0.44 (0.07,2.71) | 0.58 (0.09,3.51) | Etrol |  |  |  |  |
| 0.75 (0.21,2.69) | 0.99 (0.28,3.46) | 1.72 (0.47,6.33) | Inflix |  |  |  |
| 0.68 (0.13,3.64) | 0.90 (0.17,4.70) | 1.56 (0.29,8.49) | 0.91 (0.31,2.68) | Guselku |  |  |
| 0.44 (0.03,6.59) | 0.58 (0.04,8.58) | 1.01 (0.14,7.41) | 0.59 (0.05,6.37) | 0.65 (0.05,8.85) | Golimu |  |
| 0.93 (0.15,5.87) | 1.22 (0.20,7.60) | 2.13 (0.33,13.69) | 1.24 (0.33,4.67) | 1.36 (0.25,7.55) | 2.10 (0.14,31.95) | Placebo |

**Supplementary Table 7.** Overview of newly established and emerging drugs for UC

| Drug | Target | Advantage | Shortcoming | Drug stage | Approval state Y/N |
| --- | --- | --- | --- | --- | --- |
| Infliximab | Anti-TNF | It is similar to cyclosporine in clinical response and early colon resection rate, and the relapse rate is lower in infliximab patients compared to cyclosporine patients | There is an increased risk of severe infection associated with the use of anakinra or abatacept. | Phase III completed | Y |
| Etrolizumab | α4β7 and αEβ7 integrins | It does not penetrate the central nervous system. | Compared to placebo, it was found that it did not meet its primary endpoint as a maintenance therapy. | Phase III completed | Y |
| Adalimumab | Anti-TNF | The convenience of self-injection will make it popular for remote patients | Dose escalations were associated with substantial increases in direct drug costs | Phase III completed | Y |
| Vedolizumab | α4β7 integrin | With better clinical outcomes and lower direct medical costs | The potential risk of deadly viral encephalitis, specifically progressive multifocal leukoencephalopathy. | Phase III completed | Y |
| Ustekinumab | LI-23 | Rapid relieving effect on UC symptoms. In long-term follow-ups, many patients do not require the use of corticosteroids or other medications. Additionally, multiple meta-analyses have demonstrated the reliable clinical effectiveness and safety of this medication. | The most common adverse event reported is headache. | Phase III completed | Y |
| PF-00547659 | MadCAM-1 | Not constitutively expressed in the central nervous system. | There is limited subsequent research. | Phase II completed | N |
| Golimumab | Anti-TNF | The combination of guselkumab is superior to the single drug therapy in terms of clinical efficacy | There have been more reports of lower respiratory tract infections in adverse events. 42% of patients with a history of previous use of biologics failed to respond to treatment due to primary non-response. | Phase III completed | Y |
| Visilizumab | CD3 | There is limited subsequent research. | It is ineffective for intravenous administration of corticosteroids refractory UC | Phase I/II completed | N |
| BMS-936557 | IP-10 | There is limited subsequent research. | | Phase II completed | N |
| Basiliximab | anti-CD25 | There is limited subsequent research. | | Phase II completed | N |
| Daclizumab | anti-CD25 | There is limited subsequent research. | | Phase IIb completed | N |
| Eldelumab | IP-10 | There is limited subsequent research. | | Phase IIb completed | N |
| Cobitolimod | TLR9 | There is limited subsequent research. | | Phase IIb completed | N |
